# Genetic regulatory and biological implications of the 10q24.32 schizophrenia risk locus

**DOI:** 10.1093/brain/awac352

**Published:** 2022-09-24

**Authors:** Junyang Wang, Jiewei Liu, Shiwu Li, Xiaoyan Li, Jinfeng Yang, Xinglun Dang, Changgai Mu, Yifan Li, Kaiqin Li, Jiao Li, Rui Chen, Yixing Liu, Di Huang, Zhijun Zhang, Xiong-Jian Luo

**Affiliations:** Key Laboratory of Animal Models and Human Disease Mechanisms of the Chinese Academy of Sciences and Yunnan Province, Kunming Institute of Zoology, Chinese Academy of Sciences, Kunming, YN 650223, China; Key Laboratory of Animal Models and Human Disease Mechanisms of the Chinese Academy of Sciences and Yunnan Province, Kunming Institute of Zoology, Chinese Academy of Sciences, Kunming, YN 650223, China; Key Laboratory of Animal Models and Human Disease Mechanisms of the Chinese Academy of Sciences and Yunnan Province, Kunming Institute of Zoology, Chinese Academy of Sciences, Kunming, YN 650223, China; Key Laboratory of Animal Models and Human Disease Mechanisms of the Chinese Academy of Sciences and Yunnan Province, Kunming Institute of Zoology, Chinese Academy of Sciences, Kunming, YN 650223, China; Key Laboratory of Animal Models and Human Disease Mechanisms of the Chinese Academy of Sciences and Yunnan Province, Kunming Institute of Zoology, Chinese Academy of Sciences, Kunming, YN 650223, China; Key Laboratory of Animal Models and Human Disease Mechanisms of the Chinese Academy of Sciences and Yunnan Province, Kunming Institute of Zoology, Chinese Academy of Sciences, Kunming, YN 650223, China; Department of Neurology, Affiliated Zhongda Hospital, Southeast University, Nanjing, JS 210096, China; Key Laboratory of Animal Models and Human Disease Mechanisms of the Chinese Academy of Sciences and Yunnan Province, Kunming Institute of Zoology, Chinese Academy of Sciences, Kunming, YN 650223, China; Key Laboratory of Animal Models and Human Disease Mechanisms of the Chinese Academy of Sciences and Yunnan Province, Kunming Institute of Zoology, Chinese Academy of Sciences, Kunming, YN 650223, China; Key Laboratory of Animal Models and Human Disease Mechanisms of the Chinese Academy of Sciences and Yunnan Province, Kunming Institute of Zoology, Chinese Academy of Sciences, Kunming, YN 650223, China; Key Laboratory of Animal Models and Human Disease Mechanisms of the Chinese Academy of Sciences and Yunnan Province, Kunming Institute of Zoology, Chinese Academy of Sciences, Kunming, YN 650223, China; Key Laboratory of Animal Models and Human Disease Mechanisms of the Chinese Academy of Sciences and Yunnan Province, Kunming Institute of Zoology, Chinese Academy of Sciences, Kunming, YN 650223, China; Key Laboratory of Animal Models and Human Disease Mechanisms of the Chinese Academy of Sciences and Yunnan Province, Kunming Institute of Zoology, Chinese Academy of Sciences, Kunming, YN 650223, China; Zhongda Hospital, School of Life Sciences and Technology, Advanced Institute for Life and Health, Southeast University, Nanjing, JS 210096, China; Department of Neurology, Affiliated Zhongda Hospital, Southeast University, Nanjing, JS 210096, China; Department of Mental Health and Public Health, Faculty of Life and Health Sciences, Shenzhen Institute of Advanced Technology, Chinese Academy of Sciences, Shenzhen, GD 518055, China; Zhongda Hospital, School of Life Sciences and Technology, Advanced Institute for Life and Health, Southeast University, Nanjing, JS 210096, China; Department of Neurology, Affiliated Zhongda Hospital, Southeast University, Nanjing, JS 210096, China

**Keywords:** schizophrenia, functional risk variant, rs10786700, *SUFU*, dendritic spine density

## Abstract

Genome-wide association studies have identified 10q24.32 as a robust schizophrenia risk locus. Here we identify a regulatory variant (rs10786700) that disrupts binding of transcription factors at 10q24.32. We independently confirmed the association between rs10786700 and schizophrenia in a large Chinese cohort (*n* = 11 547) and uncovered the biological mechanism underlying this association. We found that rs10786700 resides in a super-enhancer element that exhibits dynamic activity change during the development process and that the risk allele (C) of rs10786700 conferred significant lower enhancer activity through enhancing binding affinity to repressor element-1 silencing transcription factor (REST). CRISPR-Cas9-mediated genome editing identified *SUFU* as a potential target gene by which rs10786700 might exert its risk effect on schizophrenia, as deletion of rs10786700 downregulated *SUFU* expression. We further investigated the role of *Sufu* in neurodevelopment and found that *Sufu* knockdown inhibited proliferation of neural stem cells and neurogenesis, affected molecular pathways (including neurodevelopment-related pathways, PI3K-Akt and ECM-receptor interaction signalling pathways) associated with schizophrenia and altered the density of dendritic spines. These results reveal that the functional risk single nucleotide polymorphism rs10786700 at 10q24.32 interacts with REST synergistically to regulate expression of *SUFU*, a novel schizophrenia risk gene which is involved in schizophrenia pathogenesis by affecting neurodevelopment and spine morphogenesis.

## Teaser

Genome-wide association studies have identified 10q24.32 as a robust schizophrenia risk locus. Wang *et al.* identify a regulatory variant—rs10786700—that disrupts binding of transcription factors at 10q24.32 and uncover the biological mechanism underlying the association between rs10786700 and schizophrenia.

## Introduction

Schizophrenia (SCZ) is a debilitating mental disorder with a neurodevelopmental origin.^1,2,3,4^ The lifetime prevalence of SCZ is approximately 1% in the general population.^5^ High morbidity and mortality makes SCZ a major threat to global health.^6,7^ Although SCZ imposes a tremendous economic burden on society and is a leading cause of disability,^8,9,10^ so far, therapeutic approaches for SCZ are limited and many cases do not respond to pharmacological treatments. More importantly, most antipsychotics were discovered decades ago, and these drugs exert their main therapeutic effects by antagonizing the type 2 dopaminergic receptor.^11^ Discovery of new and effective antipsychotics has proved to be extremely difficult, partly due to the unknown pathophysiology of SCZ. Identifying the risk factors involved in SCZ is the first step towards elucidating the pathophysiology of SCZ and developing new treatments.

High heritability (the heritability of SCZ was estimated as high as 81%) indicates that genetic components play important roles in the pathogenesis of SCZ.^12^ The advent of the genome-wide association study (GWAS) has provided an unprecedented opportunity to decipher the genetic basis of SCZ. To date, multiple large-scale GWASs have been performed in different continental populations and hundreds of risk loci have been reported for SCZ.^13,14,15,16^ Of note, a recent large-scale study by Trubetskoy *et al.*^17^ identified 287 risk loci for SCZ, expanding the SCZ risk loci substantially. Although these GWASs provided pivotal insights into the genetic architecture of SCZ, translating the GWAS findings into disease biology and clinical applications remains challenging. Identifying the functional (or potential causal) variants at the reported risk loci is a critical step towards elucidating the molecular mechanisms by which a risk variant confers disease risk. In our previous study,^18^ we identified 132 functional single nucleotide polymorphisms (SNPs; from the SCZ risk loci identified by GWAS) that disrupt binding of transcription factors (TFs), suggesting the potential causality of these functional SNPs. Among these TF binding-disrupting SNPs, rs10786700 showed robust association with SCZ (*P* = 9.76 × 10^−21^, 56 418 cases and 78 818 controls).^16^ Clearly, these functional SNPs could provide important genetic insights into disease susceptibility and biological implications of SCZ. Nevertheless, so far, there are no known regulatory or molecular mechanisms underlying the function of rs10786700.

To delineate the regulatory mechanisms and investigate the potential biological implications of the functional SNP rs10786700, we explored the role of rs10786700 in SCZ. We first independently confirmed that rs10786700 was associated with SCZ in the Chinese population, with the same risk allele as in European populations. We then verified the regulatory effect and functionality of rs10786700 with reporter gene and electrophoretic mobility shift assays (EMSA). We further showed that the functional variant rs10786700 is located in a super-enhancer (SE) element and regulates *SUFU* expression by interacting with repressor element-1 silencing transcription factor (REST). Of note, we demonstrated that the risk allele (C) of rs10786700 conferred lower enhancer activity through enhancing binding affinity to REST. We also found that the SE element containing rs10786700 exhibits dynamic activity change during the development process, i.e. the activity of the SE element containing rs10786700 decreases gradually as the development progresses, indicating that rs10786700 mainly exerts its regulatory effect at an early stage of neurodevelopment. Finally, we demonstrated the potential role of *SUFU* (whose expression is regulated by rs10786700) in SCZ pathogenesis using the neural stem cell and primary cortical neuron models. Our study elucidated the complex regulatory mechanisms of rs10786700 and revealed that this functional risk variant modulates the activity of the SE (where rs10786700 resides) through differential binding to EP300 and REST. Of note, this regulation is a dynamic process that mainly occurs at an early stage of neurodevelopment and this dynamic regulation led to differential expression of *SUFU* (at an early neurodevelopmental stage), which eventually confers SCZ risk by affecting neurodevelopment and dendritic spine density.

## Materials and methods

All of the reagents, kits, software and instruments used in this study are provided in Supplementary material.

### Functional genomics analysis

The procedures of functional genomics analysis have been described in our previous studies.^18^ Briefly, chromatin immunoprecipitation sequencing (ChIP-seq) data performed in brain tissues or cell lines originated from the brain of 30 TFs were downloaded from ENCODE,^19^ and binding motifs of these TFs were derived. Risk SNPs were then mapped to the derived binding motifs to investigate if different alleles of the SNPs disrupt (or affect) binding of TFs. DNase-seq and histone modification data were downloaded from ENCODE to explore if rs10786700 is located in active regulatory element.

### Genetic association analysis and meta-analysis

We investigated the genetic association between rs10786700 and SCZ in a large Chinese cohort, including 3 718 SCZ cases and 7 829 controls. All SCZ cases were carefully assessed by at least two experienced psychiatrists and all available information, including onset of symptoms, medications, illness duration, medical records and conversations with family members were evaluated thoroughly to reach a consensus diagnosis (according to the DSM-IV criteria). The mean age of cases and controls were 42 ± 15.75 and 36 ± 9.59 years, respectively. The healthy controls were recruited from local volunteers. Informed consent was obtained from all participants and this study was approved by the internal review board of Kunming Institute of Zoology. More detailed information about sample recruitments and diagnosis procedures was described in our previous studies.^20,21^ Genotyping was performed using the SNaPShot method. Detailed information about genotyping is provided in the Supplementary material. The primer sequences are provided in Supplementary Table 1. PLINK (v1.07) was used to test the association between rs10786700 and SCZ.^22^ We also examined the association between rs10786700 and SCZ in UK Biobank (571 cases and 365 476 controls) and FINNGEN (https://www.finngen.fi/en; release 5, 760 cases and 249 610 controls) datasets.^23^ We conducted a meta-analysis (using PLINK) to obtain the overall *P*-value between rs10786700 and SCZ in the combined samples.

### Cell culture

HEK293T, SK-N-SH and SH-SY5Y cells were purchased from the Cell Bank of Kunming institute of Zoology, Chinese Academy of Sciences. Mouse neural stem cells (mNSCs) and rat primary cortical neurons were isolated from embryos of wild-type mice (embryonic Day 13.5, C57BL/6) and rats (embryonic Day 18, SD), respectively. These cells were isolated as previously described.^18,19,20^ Detailed information about cell culture is provided in Supplementary material.

### Dual-luciferase reporter gene assays

Dual-luciferase reporter gene assays were performed as previously described.^18^ The primer sequences for DNA fragment cloning and point-mutation are listed in Supplementary Table 1. In brief, the 581 bp DNA fragments containing rs10786700 were cloned and inserted into the pGL3-Promoter vector (Promega, Cat. No. E1761), which was used to detect enhancer activity of the inserted sequences. HEK-293 T, SH-SY5Y and SK-N-SH cells were plated into the 96-well plates at a density of 2 × 10^4^, 6 × 10^4^ and 6.5 × 10^4^ cells/well, respectively. The recombinant vectors (150 ng) and the pRL-TK (internal control, 50 ng, Promega, Cat. No. E2241) were then co-transfected into three cell lines (HEK293T, SK-N-SH and SH-SY5Y) using the Lipofectamine^TM^ 3000 transfection reagent (Invitrogen, Cat. No. L3000-015). Forty-eight hours post-transfection, cells were lysed and luciferase activity was determined with the Dual-Luciferase® Reporter 1000 Assay System (Promega, Cat. No. E1980) on the Luminoskan Ascent chemiluminesence analyser (Thermo Scientific, Thermo Luminoskan Ascent). We also co-transferred the recombinant vectors (100 ng), the pRL-TK (50 ng) and REST overexpression plasmid (100 ng) to explore the effect of REST on the transcriptional activity of rs10786700.

### Electrophoretic mobility shift assay

EMSAs were carried out as previously described.^24^ The oligonucleotides (41 bp) containing the C or T allele of rs10786700 were synthesized and the sequences are provided in Supplementary Table 2. The synthesized oligonucleotides were labelled with biotin at the 3′ end with the EMSA Probe Biotin Labelling Kit (Beyotime, Cat. No. GS008) and annealed to form double strands. The nuclear extracts of SH-SY5Y cells were prepared using the Nuclear and Cytoplasmic Protein Extraction Kit (Beyotime, Cat. No. P0028). EMSA were performed using the Chemiluminescent EMSA Kit (Beyotime, Cat. No. GS009) according the manufacturer’s instructions. Details about EMSA are provided in the Supplementary material.

### Expression correlation analysis between *SUFU* and *EP300*

We calculated the expression correlation between *SUFU* and *EP300* in the human brain using the expression data from the LIBD dataset (*n* = 452; http://eqtl.brainseq.org/) and expression data from the study of Walker *et al.* (*n* = 219).^26,27^ Briefly, RNA sequencing was performed to quantify gene expression in the human brain in both datasets. Brain tissues (the dorsolateral prefrontal cortex) from prenatal to adulthood human individuals were included in the LIBD dataset. However, only brain tissues from prenatal subjects were included in the study of Walker *et al*.^27^ Pearson correlation coefficients were calculated and statistical tests were conducted to explore the expression correlation between *SUFU* and *EP300*.

### Knockout of the genomic region containing rs10786700

We used CRISPR-Cas9-mediated genome editing to delete the genomic region containing rs10786700. Two sgRNAs (located upstream and downstream of rs10786700) were designed using the guide design resources (https://zlab.bio/guide-design-resources; Supplementary Table 3). The designed sgRNAs were then cloned into the PX459M vector. The recombinant vector (2 μg) containing two sgRNAs was transfected into SH-SY5Y cells using Lipofectamine^TM^ 3000. The empty vectors (without sgRNA insertion) were used as the control group. Forty-eight hours post transfection, 2 μg/ml puromycin (Sigma, Cat. No. 540222) was added and cultured for 2 days to kill the untransfected cells.

### Isolation and culture of mNSCs

We isolated mNSCs according to the published protocols,^28,29^ with some minor modifications as described in our recent study.^20^ In brief, brains of mouse embryos (embryonic day 13.5, C57BL/6) were dissected under microscope and tissues from the ventricular zone (VZ) and subventricular zone (SVZ) were dissociated to obtain neural stem cells. Details about mNSC culture are provided in the Supplementary material.

### Knockdown experiments

The short hairpin RNAs (shRNAs) were designed using two online shRNA design tools (https://rnaidesigner.thermofisher.com/rnaiexpress/sort.do and https://www.sigmaaldrich.com/china-mainland/zh/life-science/functional-genomics-and-rnai/shrna/individual-genes.html). For *EP300* knockdown experiments, three pairs of shRNAs were designed for *EP300* (human) and the two pairs with high knockdown efficiency were used for further experiments. For neural stem cell experiments, six pairs of shRNAs were designed for *Sufu* (mouse) and the shRNAs with relatively higher knockdown efficiency (>50%) were used for further experiments. For dendritic spine assays, five pairs of shRNAs were designed for *Sufu* (rat) and the two shRNAs pairs with high knockdown efficiency were used for further experiments. The target regions of shRNAs are provided in Supplementary Fig. 1 and the sequences of shRNAs are provided in Supplementary Table 3. shRNAs targeting *Sufu* (mouse and rat) were inserted into pSicoR-Ef1a-mCh-puro vector (Addgene, Cat. No. 31845). Protein sequence alignment (using COBALT^30^) showed that the amino acid sequence of SUFU is highly conserved in human, mouse and rat (Supplementary Fig. 2), indicating the functional importance of SUFU. Detailed information about knockdown experiments is provided in the Supplementary material.

### Quantitative real-time PCR

Total RNA was extracted with TRIzol Reagent (Life technologies, Cat. No. 15596018) and cDNA (1 μg) was reversely transcribed using the PrimeScript RT reagent Kit with gDNA Eraser (TaKaLa, Cat. No. RR047B). Quantitative analysis of mRNA level was then performed using the TB Green® Premix Ex Taq™ II (Tli RNase H Plus; SYBR Green fluorescence, TaKaLa, Cat. No. RR820B). Relative mRNA expression levels were normalized to *ACTB* or *Actb*, and fold change was determined by the 2^−ΔΔCt^ method.^31^ All primer sequences are listed in Supplementary Table 4.

### Proliferation assay

Proliferation assays were performed as previously described.^20,25–32^ For 5-bromo-2′-deoxyuridine (BrdU) proliferation assay, mNSCs were plated into 24-well plates at a density of 10 × 10^4^ cells per well. After culturing for 48 h, BrdU (Thermo, Cat. No. MA511282) was added into medium (final concentration 10 mM) and incubated for 1 h. BrdU-positive cells were detected by immunostaining. For cell counting kit-8 (CCK-8) proliferation assays, the mNSCs were seeded into 96-well plates at a density of 1.8 × 10^4^ cells per well. After plating cells for 0, 1, 2 and 3 days, 10 μl CCK-8 (Beyotime, Cat. No. C0042) reagent were added to each well and incubated for 2 h. The absorbance at 450 nm wavelength was detected with a Spectrophotometer (BioTek).

### Differentiation of mNSCs

Differentiation assay were performed as previously described.^25,33,34^ In brief, mNSCs were plated into 24-well plates and cultured in proliferation medium for 24 h, proliferation media were then replaced with differentiation media and cultured for 3 days for spontaneous differentiation. The cells were fixed for quantification of MAP2- (a marker for mature neurons) and GFAP- (a marker for astrocytes) positive cells by immunostaining.

### Transcriptome analysis

Total RNA (from wild-type and *Sufu* knockdown mNSCs) was isolated using TRIzol Reagents (Life Technologies, Cat. No. 15596018). Transcriptome data were generated with the Illumina NovaSeq 6000 platform. DESeq2 package was used to identify the differentially expressed genes (DEGs, |fold change| >1.5, adjusted *P* < 0.05),^35^*P* values were adjusted using the Benjamini and Hochberg method.^36^ Gene Ontology (GO) enrichment analysis and Kyoto Encyclopedia of Genes and Genomes (KEGG) enrichment analysis were then performed with ClusterProfiler.^37^ Genes expressed in mNSCs were used as background genes in GO analysis.

### Analysis of the density and morphology of dendrite spines

The density and morphology of dendritic spines were analysed as previously described.^38,39,40^ Briefly, primary cortical neurons were isolated from brain tissues of embryonic rats (E17.5–E18.5, SD rats) and cultured for 14–15 days. The recombinant pSicoR-Ef1a-mCh-Puro (containing shRNAs targeting *Sufu*) and Venus plasmids (control vectors) were co-transfected into neurons at a ratio of 3:1 (a total of 3 μg) by lipofectamine^TM^ 3000. Three days after transfection, immunofluorescence staining was carried out. NeuronStudio was used for dendritic spine analyses.^40,41^ Stubby, thin and mushroom of spines of the secondary or tertiary dendrites were used for analysis. Two to three dendrites were analysed for each neuron (the length of each analysed dendrite was >50 μm).

### Immunofluorescence staining

Detailed information about immunofluorescence staining assays is provided in the Supplementary material.

### Association between rs10786700 and cognition and regional brain volumes

To explore if rs10786700 is associated with brain structure (regional brain volumes), we examined the associations between rs10786700 and regional brain volumes using the results published by Zhao *et al*.^42^ In brief, Zhao *et al*. used brain image data of 19 629 participants from the UK Biobank and conducted GWASs to investigate the associations between genetic variation and brain volumetric phenotypes. The association results were accessed from the Brain Imaging Genetics Knowledge Portal (BIG-KP; https://bigkp.org/). We also examined the association between rs10786700 and cognitive function using the data from the study by Davies *et al*.^43^ Briefly, Davies *et al*. used data from the UK Biobank to investigate the associations between genetic variation and three cognitive functions, including memory (*n* = 112 067), verbal–numerical reasoning (*n* = 36 035) and reaction time (*n* = 111 483).^43^ Detailed information about these two studies can be found in the original publications.^42,43^

### Statistical analysis

Unpaired two-tailed Student’s *t*-test was used for quantitative PCR (qPCR) experiments, proliferation and differentiation assays of mNSCs. Statistical significance was set at *P* < 0.05. Genetic association analysis and meta-analysis were performed using PLINK (chi-square test).^22^ Transcriptome differential expression analysis was analysed using DESeq2 (Wald test).^35^ Statistical significance for differentially expressed genes was set at |fold change| >1.5, adjusted *P* < 0.05.

### Data availability

The data that support the findings of this study are available in ENCODE, PsychENCODE, UCSC genome browser, SZDB, RegulomeDB, BrainSpan atlas, 3D Interaction Viewer and database (3DIV), the comprehensive human Super-Enhancer database (SEdb), UCSC cell Browser and Human Protein Atlas. These data were derived from the following resources available in the public domain: ENCODE, https://www.encodeproject.org/; PsychENCODE, http://resource.psychencode.org/; UCSC genome browser, http://genome.ucsc.edu/; SZDB, http://www.szdb.org/; RegulomeDB, https://www.regulomedb.org/; BrainSpan atlas, http://www.brainspan.org/; 3DIV, http://kobic.kr/3div/; SEdb, http://www.licpathway.net/sedb/; UCSC cell Browser, https://autism.cells.ucsc.edu; Human Protein Atlas, https://www.proteinatlas.org/; COBALT, http://www.ncbi.nlm.nih.gov/tools/cobalt/. Other data are available from the corresponding author upon reasonable request.

## Results

### rs10786700 is associated with SCZ in the Chinese population

The psychiatric genomic consortium (PGC) reported 108 SCZ risk loci in 2014.^15^ For each risk locus, PGC identified a credible set of SNPs and there is a 99% probability that the credible set of SNPs contains the causal variants. To identify the functional SNPs from the credible set of SNPs, we previously utilized a functional genomics approach to identify risk SNPs that disrupt (or affect) binding of TFs.^18^ SNP rs10786700 (the index SNP of this risk locus is rs7907645) is one of the 132 identified functional SNPs that disrupt binding of TFs.^15^ To further explore the role of these 132 TF binding-disrupting SNPs in SCZ, we examined the associations between these SNPs and SCZ in a larger SCZ GWAS (56 418 cases and 78 818 controls).^16^ SNP rs10786700 (*P* = 9.76 × 10^−21^) showed the second most significant association with SCZ among the 132 SNPs (Supplementary Table 5). Of note, although rs3131340 (*P* = 1.57 × 10^−26^) (6p21.33) showed the most significant association, considering the complex linkage disequilibrium pattern in the major histocompatibility complex region, we focused on rs10786700 in this study.

**Figure 1:**
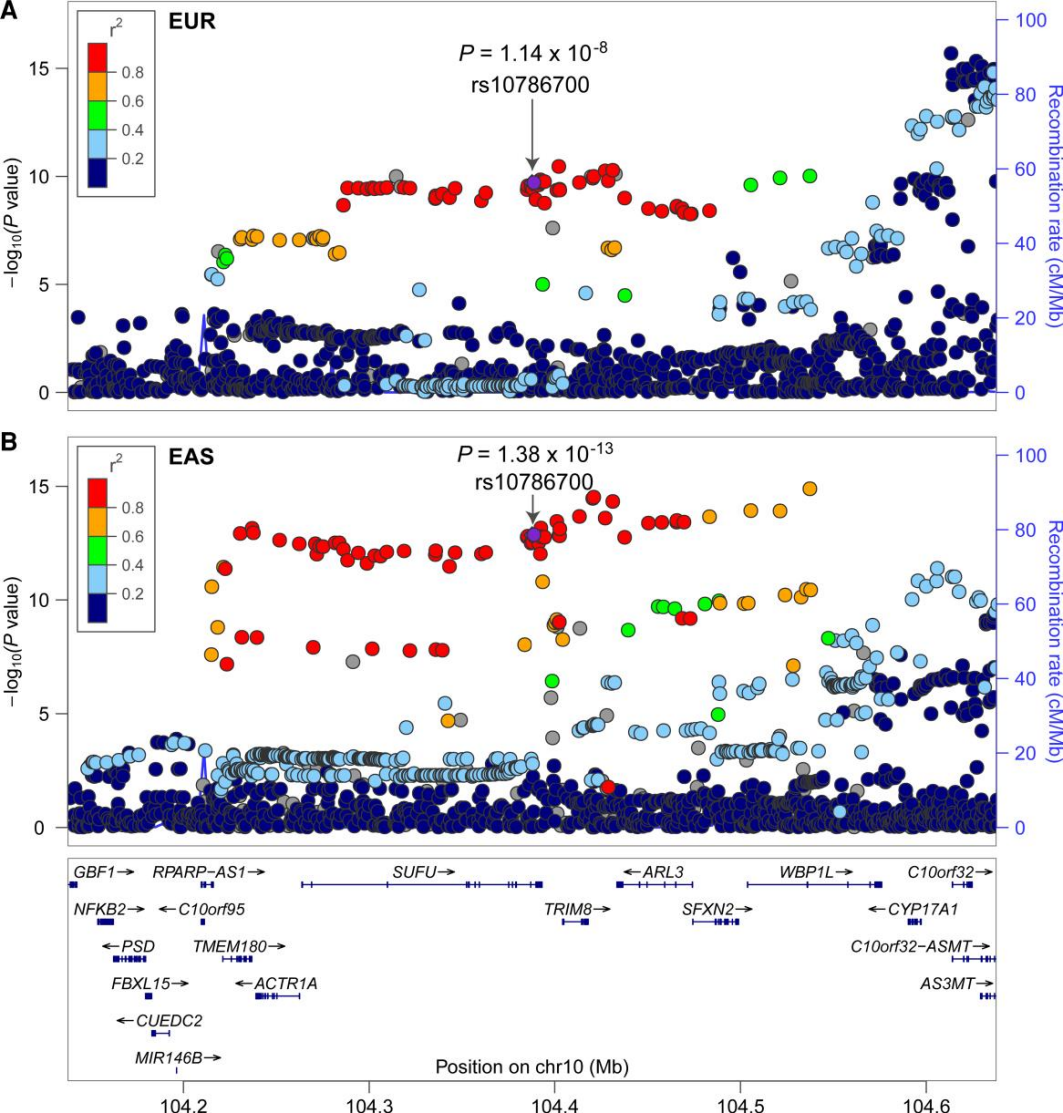
**rs10786700 shows robust association with SCZ.** (**A** and **B**) The locus zoom plots show the associations between variants near rs10786700 (500 kb) and SCZ in European and East Asian populations. rs10786700 shows genome-wide significant association with SCZ in both European and East Asian populations.

To investigate whether rs10786700 is associated with SCZ in the Chinese population, we conducted an association study and found that rs10786700 is also associated with SCZ in the Chinese population (3 718 cases and 7 829 controls; *P* = 1.56 × 10^−4^, OR_(T allele)_ = 0.897; Table 1). To further strengthen the finding of rs10786700 in association with SCZ, we conducted a trans-ancestry meta-analysis by combining association results from Europeans (including PGC, UK Biobank and FINNGEN), East Asians and our Chinese samples. Meta-analysis (66 467 cases and 701 733 controls) revealed that rs10786700 showed strong association with SCZ (*P* = 1.25 × 10^−25^, OR_(T allele)_ = 0.894; Table 1). In fact, rs10786700 is one of the few risk variants that showed genome-wide significant association with SCZ in both European (*P* = 1.14 × 10^−8^; Fig. 1A) and East Asian (*P* = 1.38 × 10^−13^; Fig. 1B) populations.^15,16^ These consistent and convergent genetic findings provide robust genetic evidence that support rs10786700 is an authentic risk variant for SCZ.

**Table 1:** rs10786700 is associated with SCZ

SNP ID	Study	Cases/controls	A_12_	OR^a^	*P*
rs10786700	This study	3718/7829	T/C	0.897	1.56 × 10^−4^
	PGC2 + EAS	56 418/78 818	T/C	0.895	9.76 × 10^−21^
	UK Biobank	571/365 476	T/C	0.874	1.32 × 10^−1^
	FINNGEN	5760/249 610	T/C	0.873	1.22 × 10^−2^
	Combined	66 467/701 733	T/C	0.894	1.25 × 10^−25^

C = C allele; T = T allele.

OR, odds ratio is based on A_1_.

### rs10786700 is a functional variant that resides in the binding motif of REST

**Figure 2:**
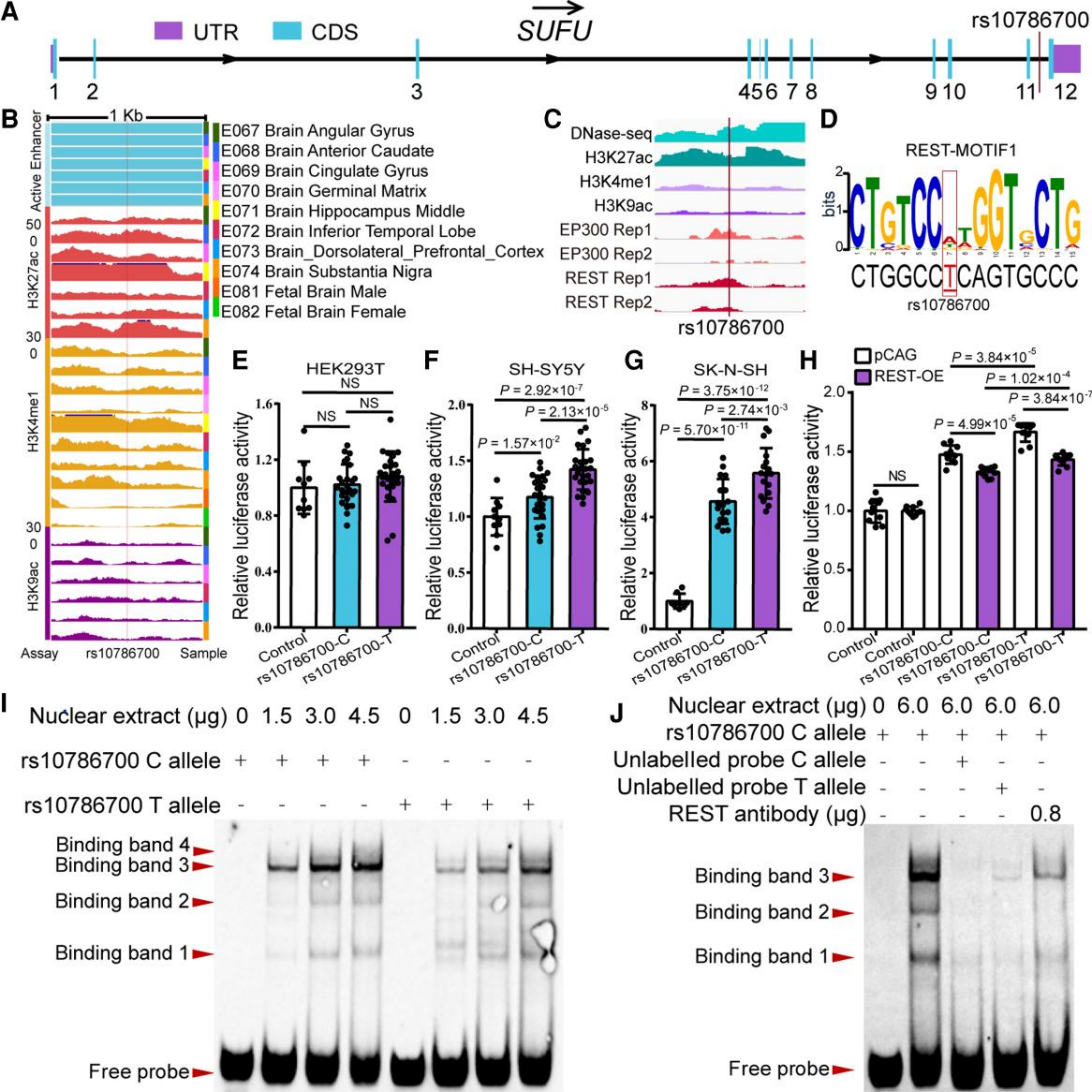
**Validation of the enhancer regulatory effect of rs10786700.** (**A**) rs10786700 is located in the last intron of the *Sufu* gene. (**B**) rs10786700 is located in an enhancer element (marked with H3K27ac, H3K4me1 and H3K9ac signals) in human foetal brain. Data were from Roadmap (http://www.roadmapepigenomics.org/). (**C**) rs10786700 is located in an open chromatin region (i.e. actively transcribed) marked with strong DNase-seq and histone H3K27ac signals in human neuroblastoma cells (SK-N-SH). (**D**) Position weight matrix (PWM) and FIMO analyses showed that rs10786700 affects REST binding in SK-N-SH cells. (**E**–**G**) Dual-luciferase reporter gene assays validated the enhancer activity of rs10786700. The genomic sequence containing rs10786700 exhibited enhancer activity compared with control vector in SH-SY5Y and SK-N-SH cells (but not HEK293T). Of note, the risk allele (C allele) of rs10786700 conferred significant lower luciferase activity compared with the T allele in SH-SY5Y and SK-N-SH cells. Values on the *y*-axis represent relative reporter gene activity (i.e. the luciferase activity of pGL3 promoter vector containing the cloned sequence relative to the pRL-TK (internal control) vector). *n* = 8 for control group, *n* = 16 for experimental groups. (**H**) REST overexpression significantly repressed the luciferase activity of the genomic fragment containing rs10786700. *n* = 8. (**I**) EMSA showed the preferential binding of nuclear extracts to C allele of rs10786700. The loaded amounts of nuclear extracts were 0, 1.5, 3.0 and 4.5 μg, respectively (from *left* to *right*). Four binding bands were detected, and the risk allele (C allele) of rs10786700 shows stronger binding affinity to nuclear extracts than the T allele for binding band 3. (**J**) Super-shift and competitive experiments for EMSA. The binding specificity of rs10786700 to transcription factors was verified by competitive experiments (using unlabelled probes). Super shift experiments were performed to detect the binding of rs10786700 to REST by adding 0.8 μg REST antibody; 6.0 μg total nuclear extracts were added to each lane. The amount of labelled probe was 50 fmol per lane. (**I** and **J**) Unpaired two-tailed Student’s *t*-test; data are presented as mean ± SD; NS, not significant.

Having confirmed that rs10786700 is robustly associated with SCZ, we next sought to elucidate the role of this risk SNP in SCZ. rs10786700 resides in the 11th intron of *SUFU* (Fig. 2A), a genomic region with multiple SNPs showed strong associations with SCZ (Fig. 1A and B).^13,15–44^ Chromatin features analysis of the genomic sequence surrounding rs10786700 showed that rs10786700 is located in an open chromatin region marked with strong DNase-seq signal in foetal human brain (Supplementary Fig. 3). In addition, histone modification data also showed that rs10786700 is located in a genomic region marked with intensive enhancer signal, including H3K27ac, H3K4me1 and H3K9ac, in foetal human brain (Fig. 2B), indicating that rs10786700 is located in an actively transcribed genomic region. In addition to the foetal human brain, we found that rs10786700 is located in an open chromatin region marked with strong DNase-Seq and H3K27ac signals in human neuroblastoma cells (SK-N-SH) (Fig. 2C). Consistent with the observations in human neuroblastoma cells, the genomic region containing rs10786700 was also marked with H3K27ac signal in other cell lines (including NHLF, NHEK, HUVEC, HSMM and H1-hESC cells; Supplementary Fig. 4). Finally, the genomic region containing rs10786700 was also marked with histone H3K27ac modification in olfactory neuroepithelium.^45^ ChIP-seq data further showed that REST could bind to the genomic sequence containing rs10786700 (Fig. 2C) and binding motif analysis revealed that different alleles of rs10786700 affect REST binding (Fig. 2D). These consistent results suggested that rs10786700 is a functional SNP that is located in an active enhancer element in the human brain and neuronal cells.

To further test the functionality of rs10786700, we performed dual-luciferase reporter assays. In line with the histone-modification data, we validated the enhancer activity of the genomic region containing rs10786700 in SH-SY5Y and SK-N-SH cells (but not in the HEK293T cell line; Fig. 2E–G). Interestingly, we found that the luciferase activity of C (risk) allele was significantly lower than that of T allele (Fig. 2F and G). To further investigate if the enhancer activity of the genomic sequence containing rs10786700 is regulated by REST, we co-transfected the recombinant vector (containing rs10786700) and REST overexpression vector. REST overexpression decreased the enhancer activity of the genomic region containing rs10786700 significantly (Fig. 2H), indicating that rs10786700 and REST act synergistically to regulate enhancer activity. We next performed EMSA to test whether different alleles of rs10786700 affect REST binding. EMSA detected four binding bands, indicating that the 41 bp DNA fragment (centred at rs10786700) could interact with nuclear extracts. EMSA also showed that the C risk allele of rs10786700 exhibited stronger binding affinity than the non-risk T allele for binding band 3 (Fig. 2I). Super-shift assays validated that REST is one of the TFs that bind rs10786700, as binding band 3 became weaker after the addition of REST antibody (Fig. 2J). Collectively, these results indicated that rs10786700 is a functional SNP that regulates the enhancer activity of the genomic sequence it located (by affecting REST binding).

### rs10786700 resides in a super-enhancer which exhibits dynamic activity change in development

**Figure 3:**
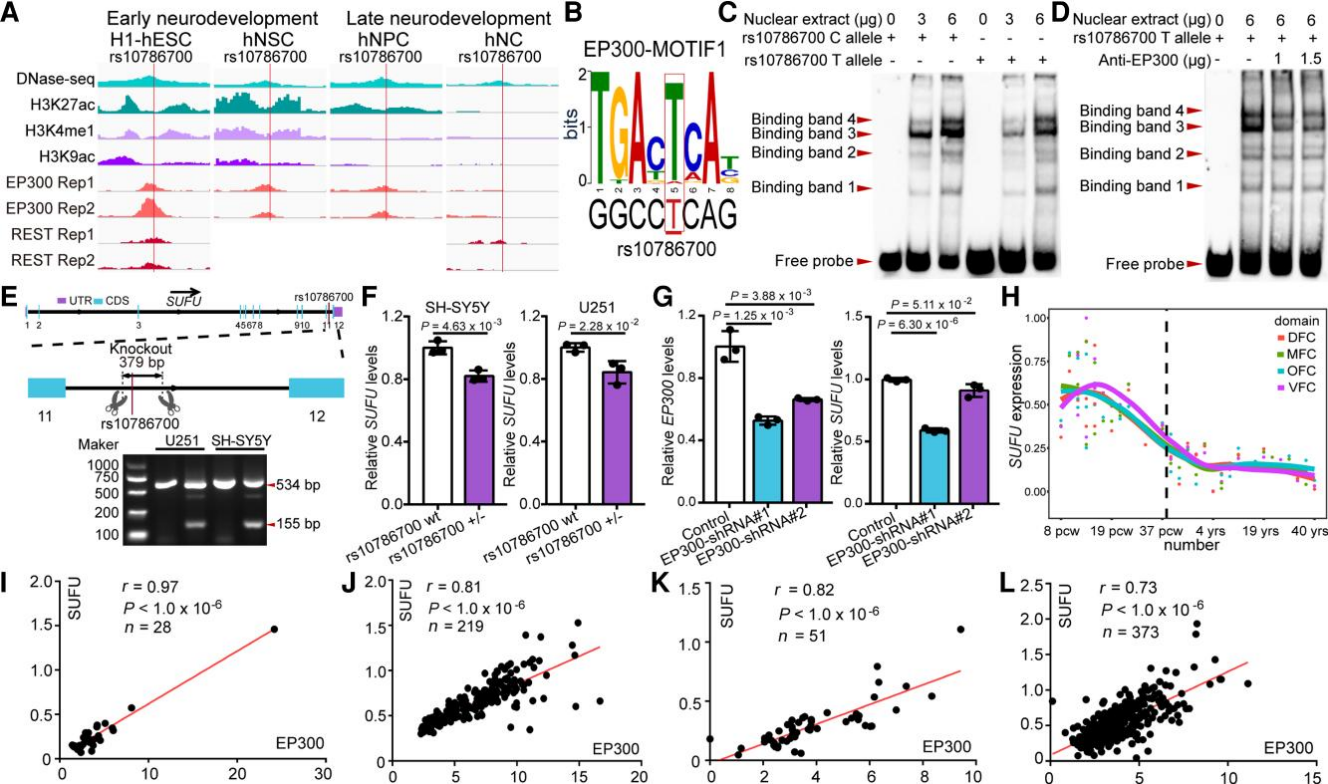
**rs10786700 regulates *SUFU* expression by dynamically modulating the activity of the SE in neurodevelopment**. (**A**) DNase-seq and histone modifications for genomic region surrounding rs10786700 (1 Mb centred on rs10786700) showed that rs10786700 is located in an actively transcribed genomic region. Of note, ChIP-Seq data showed that EP300 and REST bind to the genomic sequence containing rs10786700 in a development-dependent manner. In early neurodevelopment stage, the ChIP-seq signals of EP300 and REST were high. As development progresses, the ChIP-seq signals decrease gradually. (**B**) Position weight matrix (PWM) and FIMO analyses showed that rs10786700 disrupts binding of EP300 in SK-N-SH cells. (**C**) The amounts of nuclear extracts were 0, 3 and 6 μg, respectively (from *left* to *right*). Four binding bands were observed. The risk allele (C allele) of rs10786700 showed stronger binding affinity to nuclear extracts than the T allele for binding band 3. (**D**) Super-shift experiments validated the binding of rs10786700 to EP300. (**E**) A 379 bp fragment containing rs10786700 (located in the 11th intron of *SUFU* gene) was knocked out by CRISPR/Cas9-mediated editing in SH-SY5Y and U251 cells. (**F**) qPCR showed that rs10786700 knock-out resulted in significant downregulation of *SUFU* expression in both SH-SY5Y and U251 cells. (**G**) qPCR validation of *SUFU* expression in EP300 knocked-down SH-SY5Y cells. *n* = 3 for **F** and **G**. Unpaired two-tailed Student’s *t*-test; data are presented as mean ± SD. (**H**) Expression patterns of *SUFU* in the developing and adult human frontal cortex. Expression level of *SUFU* across the entire developing stages (from 8 pcw to 40 years) were depicted in different areas of the frontal cortex. The expression data (42 human subjects) were from BrainSpan (http://www.brainspan.org/).^50^ Pcw, post-conception weeks; yrs, years; DFC, dorsolateral prefrontal cortex; MFC, medial prefrontal cortex; OFC, orbital prefrontal cortex; VFC, ventrolateral prefrontal cortex. (**I**–**L**) The Pearson expression correlations between *SUFU* and *EP300* in the human brain. (**I**) Correlations in prenatal human brain (LIBD dataset). (**J**) Correlations in prenatal human brain (expression data from Walker *et* al.^27^). (**K**) Correlations in childhood human brain. (**L**) Correlations in adulthood human brain. Sample group: prenatal (age < 0), child (0 < age < 18), adult (age > 18), non-prenatal (age > 0).

To further investigate the effects and regulatory mechanisms of rs10786700 in SCZ, we examined the chromatin characteristics of the genomic sequence surrounding rs10786700 at several different developmental stages. Interestingly, we found that rs10786700 is located in a SE (marked with EP300 and H3K27ac) at early developmental stages, including H1 human embryonic stem cell (H1-hESC) and H1-derived neuronal progenitor cell (H1-hNPC; Supplementary Fig. 5A and B).^46,47^ However, SE activity disappeared in the dorsolateral prefrontal cortex (PFC) and hippocampus (HIP) (Supplementary Fig. 5C and D; data were from the 3D Interaction Viewer and database, 3DIV).^48^ Further interrogation using the comprehensive human Super-Enhancer database (SEdb)^49^ showed consistent results (i.e. rs10786700 is located in SE elements in hESC, hNPC and neuroblastoma cells, but not in the PFC, HIP, astrocyte and neural cells (NC); Supplementary Table 6). In addition, the genomic region containing rs10786700 was marked with EP300 binding signals in H1-hEST, human neural stem cell (hNSC), SK-N-SH and H1-hNPC, but not in NC (Fig. 3A, data were from the ENCODE portal).^19^ These data suggest that rs10786700 is located in a SE that exhibits dynamic activity change in development. That is, rs10786700 is located in a SE with strong EP300 and H3K27ac signals at early neurodevelopmental stage. As neurodevelopment progresses, the binding of EP300 to rs10786700 decreases (Fig. 3A), which inactivates the regulatory activity of the SE element gradually. Of note, the activity of the SE containing rs10786700 was completely lost in highly differentiated brain tissues (Supplementary Fig. 5 and Supplementary Table 6). Binding motif analysis revealed that rs10786700 affected EP300 binding (Fig. 3B). To further ascertain whether rs10786700 affects EP300 binding, we conducted EMSA and found that binding bands 3 and 4 were significantly weakened after the addition of EP300 antibody (Fig. 3C and D). These results indicate that both EP300 and REST could bind to the genomic sequence containing rs10786700. Taken together, these data indicate that rs10786700 dynamically regulates the activity of the SE (surrounding rs10786700) through affecting EP300 and REST binding. These results suggest that rs10786700 may exert its main effects at early neurodevelopmental stage by regulating expression of its target gene (or genes).

### rs10786700 regulates *SUFU* expression by interacting with REST and EP300

Having demonstrated the functionality and temporal regulation characteristics of rs10786700, we next sought to identify the potential target gene (or genes) by which rs10786700 exerts its biological effect on SCZ. To this end, we knocked out a 379 bp genomic sequence containing rs10786700 (located in the 11th intron of the *SUFU* gene) using CRISPR/Cas9-mediated genome editing (Fig. 3E). PCR verified that the 379 bp DNA sequence containing rs10786700 was successfully deleted in U251 and SH-SY5Y cells (Fig. 3E). We then measured the expression change of five genes (including *TMEM180*, *ACTR1A*, *SUFU*, *TRIM8* and *ARL3*) near rs10786700 (Fig. 1B) using qPCR. We found that rs10786700 deletion resulted in significant downregulation of *SUFU* and *TRIM8* in SH-SY5Y and U251 cells (Fig. 3F and Supplementary Fig. 6A). However, *TMEM180* and *ACTR1A* were downregulated in only one cell type (Supplementary Fig. 6B and C), and the expression of *ARL3* did not change in either cell type (Supplementary Fig. 6D). These results suggest that *SUFU* and *TRIM8* are two promising candidates for rs10786700, as these two genes are located nearest to rs10786700 and deletion of the genomic sequence containing rs10786700 altered the expression of these two genes in neuroblastoma (SH-SY5Y) and glioma (U251) cell lines.

To further investigate if these genes were regulated by EP300, we knocked-down *EP300* in SH-SY5Y and examined the expression change of genes near rs10786700. *EP300* knockdown did not affect *TRIM8* expression (Supplementary Fig. 6E); however, the expression of the other four genes (*SUFU*, *TMEM180*, *ACTR1A* and *ARL3*) was downregulated significantly (Fig. 3G, Supplementary Fig. 6E). These results indicate the regulation effect of EP300 on *SUFU*, *TMEM180*, *ACTR1A* and *ARL3*.

We next examined the long-range chromatin interactions between rs10786700 and nearby genes and found that rs10786700 mainly interacts with genes in the same topological associated domain (TAD) region (Supplementary Fig. 5). Of note, rs10786700 has the highest interaction frequency with the *SUFU* gene (Supplementary Fig. 5), and rs10786700 also interacts with the promoter of *SUFU* (including upstream regulatory region and transcription start site). Consistent with these results, *SUFU* also exhibits dynamic expression change during development (Fig. 3H). *SUFU* expression level was high at early neurodevelopmental stages; as neurodevelopment progresses, *SUFU* expression level gradually decreases (Fig. 3H, data from the BrainSpan atlas, http://www.brainspan.org/).^50^ To further explore if *SUFU* expression was associated with EP300 (binding to DNA sequence was affected by rs10786700) in the human brain, we conducted Pearson’s correlation analysis. The results showed that the expression level of *EP300* was significantly correlated with *SUFU* in the human brain (Fig. 3I–L). Of note, the correlation strength also showed a development stage-dependent manner, i.e. EP300 exhibited stronger correlations with *SUFU* expression at an early developmental stage (prenatal stage; Fig. 3I and J) than at childhood (Fig. 3K) and adult stages (Fig. 3L). Collectively, these results indicated that rs10786700 may regulate *SUFU* expression by interacting with the regulatory element of *SUFU*.

As rs10786700 affects binding of REST, we next investigated the synergistic effect of rs10786700 and REST on transcription regulation. REST overexpression significantly repressed the transcription activity of the genomic fragment containing rs10786700 (Fig. 2H), indicating that rs10786700 and REST act synergistically to regulate the transcription activity of the genomic sequence containing rs10786700. Collectively, these convergent lines of evidence suggest that rs10786700 regulates the expression of *SUFU* by affecting REST binding, and the C (risk) allele of rs10786700 was associated lower *SUFU* expression by enhancing REST binding affinity (which in turn decreases the activity of the enhancer element it located).

### *Sufu* regulates proliferation and differentiation of mNSCs

**Figure 4:**
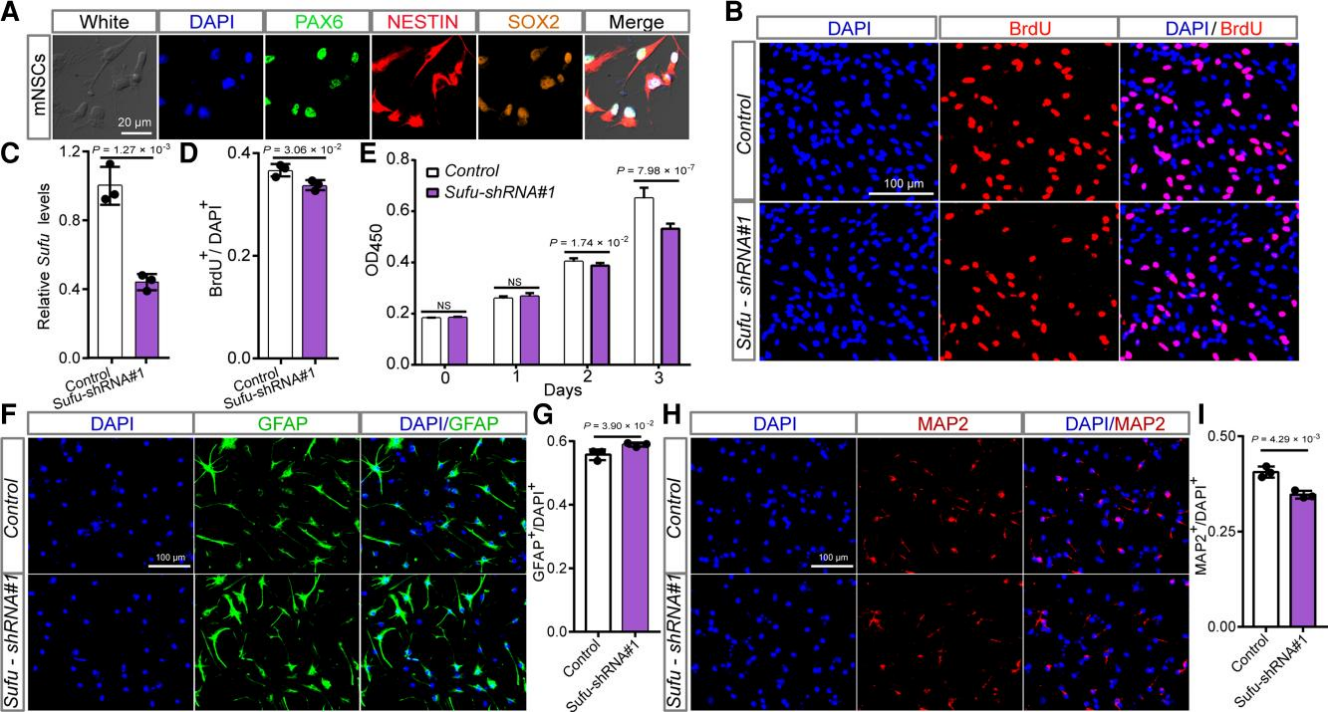
***Sufu* knockdown inhibits proliferation and neurogenesis of mNSCs.** (**A**) Immunofluorescence staining results of three well-characterized markers (SOX2, PAX6 and NESTIN) for neural stem cells confirmed the identity of the isolated mNSCs. (**B**) Immunofluorescence staining for BrdU incorporation assay. Red indicates BrdU-positive cells undergoing DNA amplification and DAPI was used to stain the nucleus (blue). (**C**) *Sufu* expression was efficiently knocked-down by the shRNA in mNSCs. (**D**) The quantification results of the BrdU incorporation assay. (**E**) The results of CCK-8 assay. Data were collected at 0, 1, 2 and 3 days after plating. (**F**) Immunofluorescence staining images for astrocyte cells (GFAP-positive cells) differentiated from mNSCs. (**G**) Quantification data for the ratio of GFAP-positive cells. *Sufu* knockdown led to a significant increase of GFAP-positive cells. (**H**) Immunofluorescence staining images for mature neurons (MAP2-positive cells) differentiated from mNSCs. (**I**) Quantification data for the percentage of MAP2-positive cells in total cells. *Sufu* knockdown resulted in a significant decrease of MAP-positive cells. *n* = 3 for **B**–**D**, **F**–**I**; *n* = 9 for **E**. mNSCs, mouse neural stem cells. Unpaired two-tailed Student’s *t*-test; data are presented as mean ± SD. NS, not significant.

Our results suggested that rs10786700 confers SCZ risk through regulating *SUFU* expression. We thus explored the potential role of *SUFU* in SCZ pathogenesis. *SUFU* expression level in the human brain (i.e. higher expression at early neurodevelopmental stage) is concordant with the dynamically changed activity of the SE containing rs10786700 (Fig. 3H), suggesting that *SUFU* may have a pivotal role in neurodevelopment. We thus investigated the effect of *Sufu* (the human *SUFU* orthologue) on neurodevelopment using the mNSCs model, a model that was widely used to explore the potential pathogenesis of SCZ.^51,52,53,54^ Immunostaining co-labelling with three well-characterized markers (PAX6, SOX2 and NESTIN) for neural stem cells confirmed that the isolated cells were NSCs (Fig. 4A). We then knocked-down *Sufu* in mNSCs and conducted BrdU and CCK-8 proliferation assays. Both BrdU and CCK-8 assays showed that *Sufu* knockdown inhibited proliferation of mNSCs significantly (Fig. 4B–E), indicating the important role of *Sufu* in NSC proliferation.

In addition to proliferation, we also investigated the role of *Sufu* in neural differentiation, an important process for neurodevelopment (many morphological and functional distinct cell types—including neurons, astrocytes and oligodendrocytes—were generated in this process). Immunofluorescence results showed that *Sufu* knockdown led to significant increase of the ratio of GFAP- (a marker for astrocytes) positive cells compared with controls (Fig. 4F and G). In contrast, the ratio of MAP2- (a marker for mature neurons) positive cells was significantly decreased in *Sufu* knockdown mNSCs (compared to controls; Fig. 4H and I). qPCR also revealed similar results (i.e. *Sufu* knockdown resulted in significant upregulation of *Gfap* mRNA expression level and downregulation of *Tuj1*, a marker for newly generated immature post-mitotic neurons; Supplementary Fig. 7). Overall, these findings demonstrate the pivotal role of *Sufu* in regulating proliferation and differentiation of NSCs.

### *Sufu* regulates neurodevelopmental-related pathways and signalling pathways associated with SCZ

**Figure 5:**
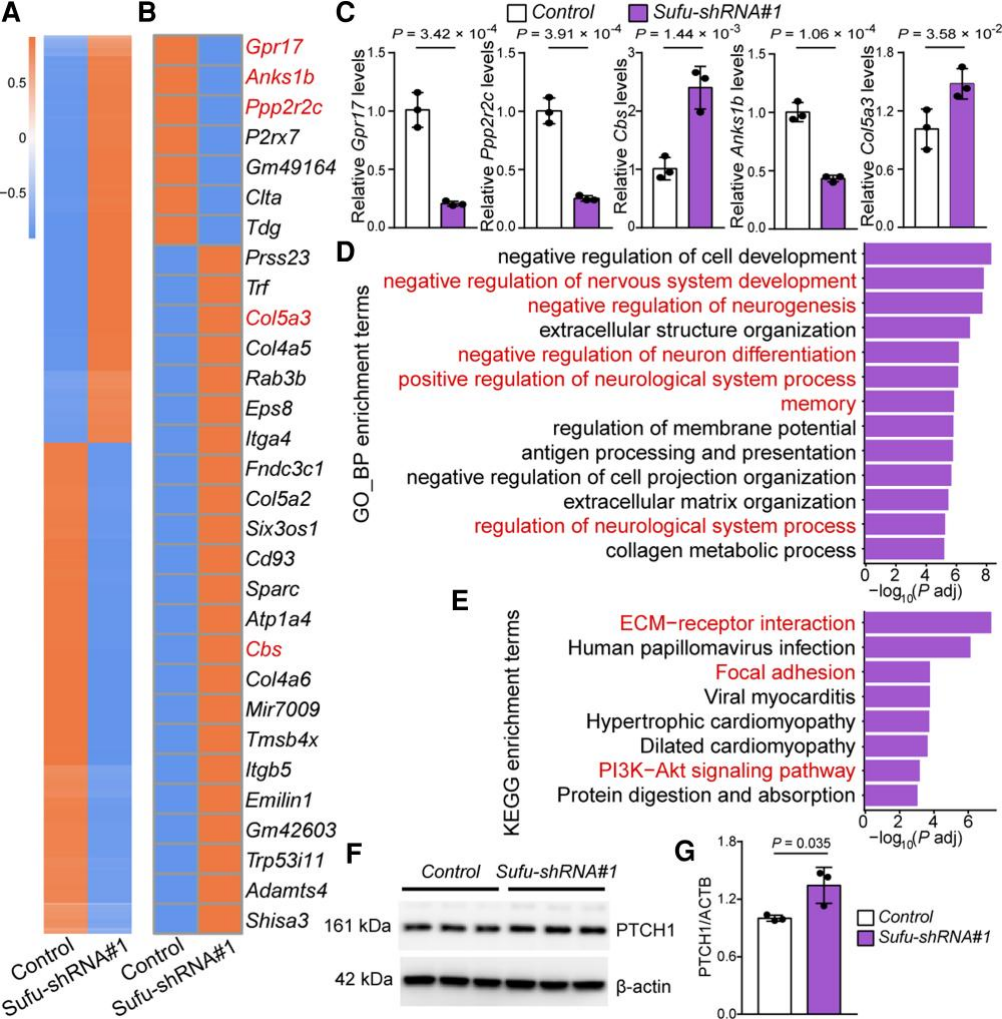
***Sufu* regulates SCZ-associated biological processes and signalling pathways.** (**A**) Expression heat map of 860 DEGs identified in *Sufu* knockdown mNSCs compared with control mNSCs. (**B**) Heat map plot of the top 30 DEGs. Five genes (marked by red colour), including *Gpr17*, *Anks1b*, *Ppp2r2s*, *Col5a3* and *Cbs*, were selected for qPCR verification (**C**). (**D**) GO analysis based on biological process for the 860 DEGs. Terms marked in red indicate schizophrenia-associated biological processes. (**E**) KEGG analysis of the 860 DEGs. Terms marked in red indicate schizophrenia-associated signalling pathways. (**F**, **G**) Validation of PTCH1 expression in *Sufu* knockdown mNSCs with Western blot. *n* = 3 for **C** and **G**; unpaired two-tailed Student’s *t*-test; data are presented as mean ± SD.

To further explore the molecular pathways regulated by *Sufu*, we performed transcriptome analysis using RNA sequencing (RNA-seq). We identified 860 DEGs (|fold change| >1.5, adjusted *P* < 0.05, Fig. 5A). Among them, 470 genes were downregulated and 390 genes were upregulated in *Sufu* knockdown mNSCs (Fig. 5A). We validated the RNA-seq results by quantifying five genes (*Grp17*, *Anks1b*, *Ppp2r2c*, *Col5a3* and *Cbs*, selected from the top 30 of DEGs) using qPCR (Fig. 5B and C). GO analysis based on biological process showed that the DEGs were mainly enriched in neurodevelopmental processes, including regulation of nervous system development, neurogenesis and nervous system process and synaptic transmission processes (Fig. 5D). In addition, the DEGs were also enriched in memory, a pivotal cognitive process that was impaired in SCZ (Fig. 5D).^55,56^ Finally, KEGG analysis revealed significant enrichments of DEGs in ECM–receptor interaction, PI3K-Akt and focal adhesion signalling pathways (Fig. 5E). Of note, these pathways have been reported to be dysregulated in SCZ.^57,58,59,60,61,62,63,64^ In summary, these results demonstrate that *Sufu* regulates neurodevelopmental processes and suggest *SUFU* may contribute to SCZ risk by affecting these SCZ-associated biological processes (such as neurodevelopment) and signalling pathways (such as including ECM–receptor interaction,^57,58,59^ focal adhesion and PI3K-Akt signalling pathway).^60,61,62,63,64^

### *Sufu* regulates the density of dendritic spines

**Figure 6:**
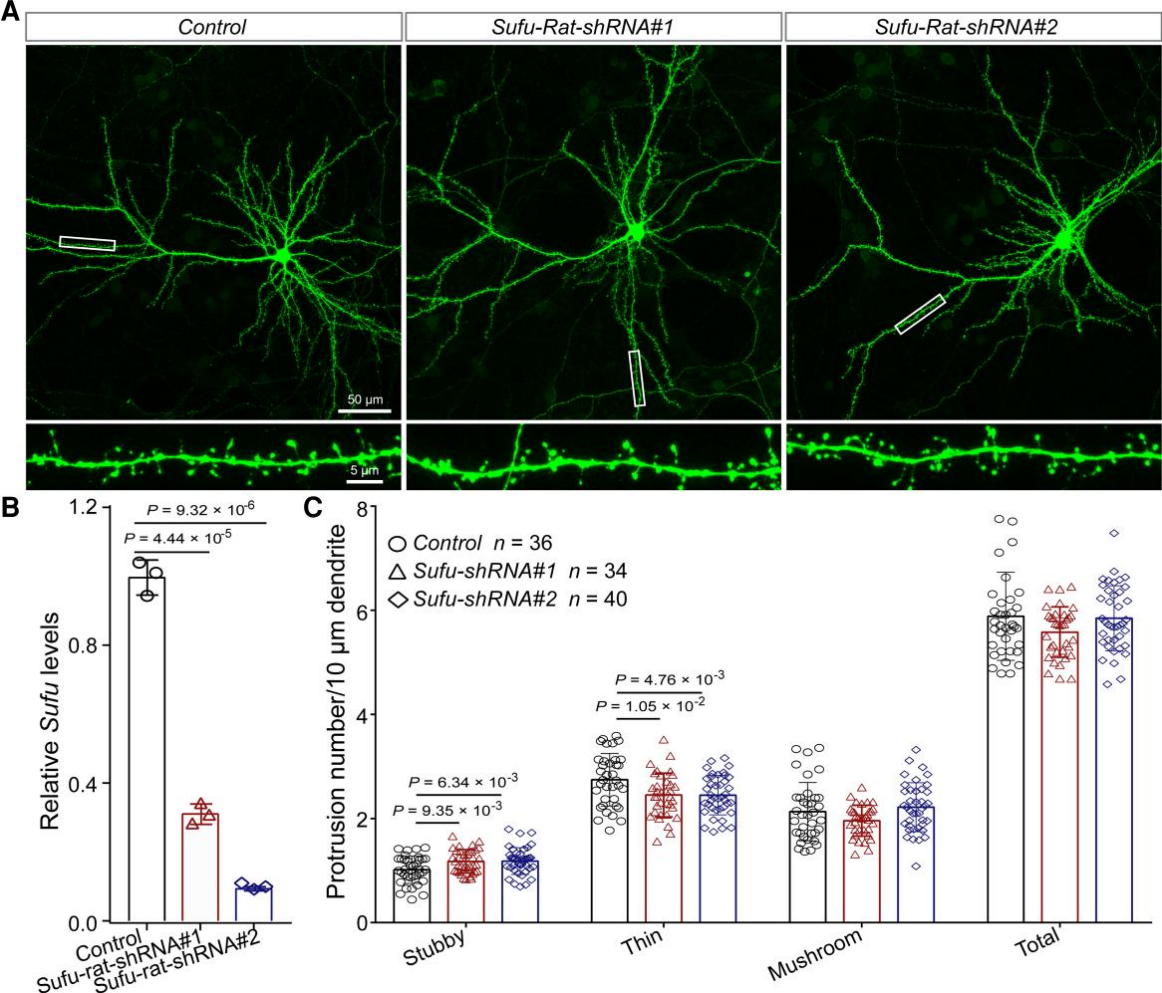
***Sufu* regulates dendritic spine density.** (**A**) Representative immunofluorescence staining images for the density and morphology of dendritic spines in *Sufu* knockdown neuron compared with control neuron. (**B**) *Sufu* expression in rat primary neurons was significantly knocked-down by shRNAs. (**C**) The result of dendritic spine density analysis for stubby, thin and mushroom spines. *n* = 36 for control, *n* = 34 for Sufu-Rat-shRNA#1, *n* = 40 for Sufu-Rat-shRNA#2. Unpaired two-tailed Student’s *t*-test; data are presented as mean ± SD.

Although the pathophysiology of SCZ remains largely unknown so far, accumulating evidence supports the dendritic spine pathology of SCZ.^65,66,67,68,69^ Intriguingly, GO analysis based on cell components (CC) showed that the DEGs were significantly enriched in synapse component, including the postsynaptic component (Supplementary Fig. 8), implying the potential role of *Sufu* in dendritic spine morphogenesis. Single-cell RNA sequencing data also showed that *SUFU* is mainly expressed in neurons, especially in excitatory neurons (L2/3, L4, L5/6 and L5/6-CC; Supplementary Fig. 9, data were from the UCSC cell Browser, https://autism.cells.ucsc.edu).^70^ To further study the potential role of *Sufu* in SCZ, we investigated the effect of *Sufu* knockdown on dendritic spine density, one of the most well-characterized pathophysiological alternations in SCZ. *Sufu* knockdown in rat primary neurons did not affect the density of mushroom spines (a type of mature dendritic spines; Fig. 6A–C). Nevertheless, the density of stubby spines (a type of mature dendritic spines) was significantly increased and the density of thin spines (immature dendritic spines) significantly decreased in *Sufu* knockdown neurons compared with controls (Fig. 6A–C). These findings indicate that *Sufu* plays a role in the morphogenesis of dendritic spines, suggesting that *Sufu* may confer SCZ risk through affecting dendritic spine morphology. These results also provide further evidence that support the dendritic spine pathology of SCZ.

## Discussion

rs10786700 is located in 10q24.32, a risk locus which has been repeatedly reported to be associated with SCZ.^15,16,17^ Several previous studies have explored the underlying risk variants and genes responding for the association. For example, Duarte *et al*. found that the schizophrenia risk variant rs11191419 at this locus is associated with the expression of *BORCS7*, *AS3MT* and *NT5C2* in the human brain.^73^ Li *et al*. also found that multiple polymorphic variants (include a human-specific variable number of tandem repeats (VNTR)) in this risk locus were associated with the expression of *AS3MT* and *BORCS7*.^74^ Interestingly, Li *et al*. showed that a specific *AS3MT* isoform (which lacks exon 2 and 3, *AS3MT*^d2d3^) may have a role in SCZ.^38^ Besides, Ding *et al*. found that the risk allele (A allele) of rs5011218 promotes *TRIM8* expression by increasing the binding affinity of POU3F2, and *TRIM8* knockdown promotes human neural progenitor cell (NPC) proliferation, inhibits neurogenesis and impairs excitatory synaptic transmission in NPC-derived neurons.^75^ Another candidate risk gene in this locus is *ARL3*, a schizophrenia susceptibility gene shared by Chinese and European populations.^18–76^ Finally, a recent study identified *TMEM180* (located at 10q24.32) as a schizophrenia risk gene through transcriptome-wide association studies (TWAS) and Mendelian randomization analysis (SMR). *TMEM180* was downregulated in peripheral blood of schizophrenia patients and affects proliferation and neurogenesis of mNSCs.^25^ These results indicate that the 10q24.32 risk locus may harbour multiple schizophrenia risk genes and variants.

**Figure 7:**
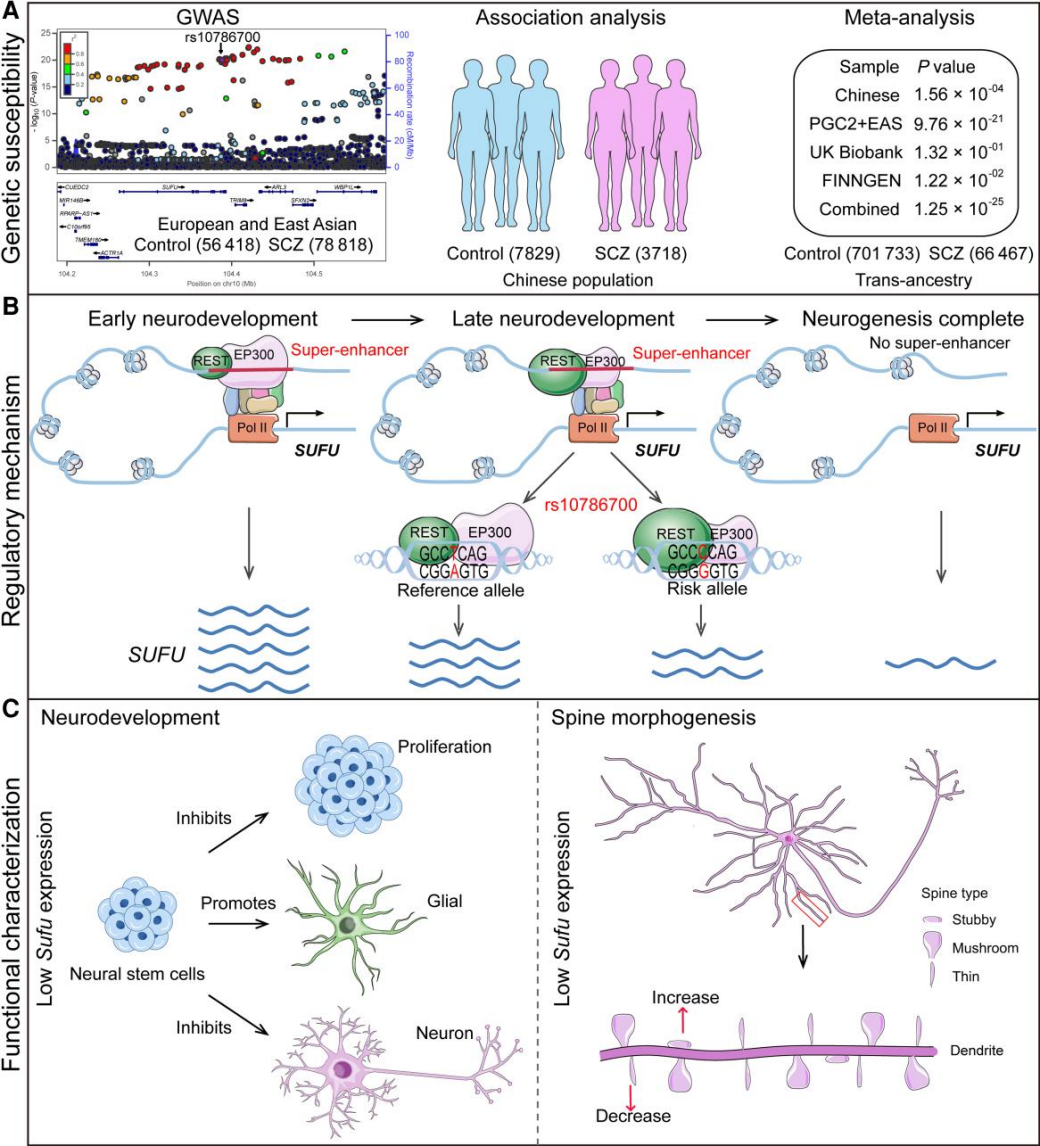
**The working model of rs10786700 in SCZ pathogenesis.** (**A**) rs10786700 is located at 10q24.32 schizophrenia risk locus, a genomic region with multiple SNPs showing robust associations with SCZ. We confirmed the genetic association between rs10786700 and SCZ in a large Chinese cohort (3 718 cases, 7 829 controls; *P* = 1.56 × 10^−04^). Trans-ancestry meta-analysis indicated that rs10786700 was robustly associated with SCZ (66 467 cases, 701 733 controls; *P* = 1.25 × 10^−25^). (**B**) Molecular regulatory mechanisms of rs10786700. rs10786700 modulates the activity of the SE (where rs10786700 is located) by dynamically binding EP300 and REST during neurodevelopment. During early neurodevelopmental stages, the genomic region containing rs10786700 exhibits strong SE activity and rs10786700 regulates *SUFU* expression through affecting REST binding. As development progresses, the activity of the SE containing rs10786700 decreased gradually. The risk allele (C allele) of rs107867000 conferred significant lower *SUFU* expression by enhancing REST binding. (**C**) The function of *Sufu* in the CNS and the potential role of *Sufu* in SCZ pathogenesis. As a negative regulator of sonic hedgehog signalling pathway, *Sufu* plays an important role in brain development. Lower *Sufu* expression inhibits proliferation of mNSCs, promotes gliogenesis and represses neurogenesis. Of note, *Sufu* knockdown also affected dendritic spine density. Collectively, these results indicated that the functional variant rs10786700 may confer risk of SCZ by dynamically regulating expression of *SUFU* in the developing human brain, and dysregulation of *SUFU* contributes to SCZ pathogenesis by affecting neurodevelopment and spine morphogenesis (two characteristic features of schizophrenia pathophysiology).

In this study, we identified rs10786700 as one of potential pathogenic SNPs for SCZ at the 10q24.32 risk locus and we demonstrated the molecular regulation and biological mechanisms of rs10786700 in SCZ (Fig. 7). First, we provide lines of robust evidence that support rs10786700 being an authentic risk variant for SCZ (Fig. 7A). Considering rs10786700 is one of the few risk variants that achieve the genome-wide significance (GWS) level in both East Asian (*P* = 1.14 × 10^−13^) and European populations (*P* = 1.38 × 10^−08^), delineation of the function of this variant will provide important insights into SCZ pathogenesis. Second, we validated the regulatory effect of rs10786700. We demonstrated that rs10786700 resides in the REST binding motif and regulates the activity of the enhancer element by affecting binding affinity to REST. Third, we showed that rs10786700 is located in an SE that exhibits dynamically activity change during neurodevelopment, and we identified *SUFU* as the potential target gene regulated by rs10786700. The binding of EP300 to the enhancer element containing rs10786700 is strong at early developmental stages, indicating the high enhancer activity of this regulatory element. As development progresses, the activity of the SE containing rs10786700 decreases gradually. Consistently, *SUFU* expression level is concordant with the dynamic activity change of the enhancer element containing rs10786700 (Fig. 3). That is, the *SUFU* expression level shows a high degree of concordance with the regulatory activity of the SE containing rs10786700. Fourth, we revealed how different alleles of rs10786700 affect enhancer activity and *SUFU* expression. The risk allele (C allele) of rs10786700 exhibits stronger binding affinity to REST (RE1 silencing transcription factor) than does the T allele. Fifth, to further explore the potential role of *SUFU* in SCZ pathogenesis, we investigated the function of *Sufu* in neurodevelopment and dendritic spine morphogenesis. We found that *Sufu* regulates proliferation, differentiation and dendritic spine morphogenesis, implying that *SUFU* may contribute to SCZ risk by affecting neurodevelopment. Finally, transcriptome analysis showed that *Sufu* knockdown affects biological processes and pathways associated with SCZ, including negative regulation of nervous system development, synaptic transmission, ECM–receptor interaction,^57,58,59^ focal adhesion and PI3K-Akt signalling pathways.^60,61,62,63,64^ These results not only elucidated the molecular regulatory mechanisms of rs10786700, but also revealed the potential role of the target gene *SUFU* located in this risk locus in SCZ pathogenesis.

We also explored whether rs10786700 was associated with other psychiatric disorders and found that it is associated with bipolar disorder (BIP, 41 917 cases and 371 549 controls; *P* = 4.08 × 10^−5^),^77^ but is not associated with autism spectrum disorder (ASD),^78^ major depressive disorder (MDD),^79^ anxious disorder,^80^ posttraumatic stress disorder (PTSD),^81^ attention deficit/hyperactivity disorder (ADHD) and panic disorder (Supplementary Fig. 10).^82,83^ These associations indicate that rs10786700 may be a risk variant for BIP and SCZ.^84,85^

One of the novel findings of this study is that we found that rs10786700, a functional variant showing genome-wide significant association with SCZ in both East Asian and European populations, is located in an SE of *SUFU*. SEs consist of clusters of enhancers occupied by high-density transcriptional regulators and they are usually marked by the strong signal of EP300^86^ or the marks of H3K27ac and H3K4me1.^46,47^ SEs have strong transcriptional activation ability^86^ and are involved in cell differentiation and tumour pathogenesis.^47,48^ SEs play important roles in regulation of gene expression, especially in the process of development. SEs have strong cell-type specificity and different cell types have a different SE landscape and SE-associated TF networks. The trait-associated SNPs were mainly enriched in SEs rather than in typical enhancers.^90^ Previous studies have revealed the important roles of SEs in complex diseases and developmental disorders,^90,91^ including Type 1 diabetes, rheumatoid arthritis and Alzheimer’s disease.^90^ The genetic variation in SEs can affect disease risk by regulating gene expression. For example, rs2168101, a polymorphism within an SE element located in the first intron of *LMO1*, influences neuroblastoma susceptibility by changing GATA transcription factor binding and regulating *LMO1* expression.^92^ rs539846, which resides in the SE (located in intron 3) of *BMF*, influences chronic lymphocytic leukaemia susceptibility through differential RELA binding and modulation of *BMF* expression.^93^ Despite the important roles of genetic variants in SEs in complex diseases, so far, the role of risk variants located in SEs in SCZ pathogenesis is unclear. In this study, we showed that the risk variant rs10786700 in SE has a pivotal role in SCZ. We demonstrated that rs10786700, a SNP that is located in an SE in the last intron of *SUFU*, may confer SCZ risk by regulating the expression of *SUFU* (through modulating REST binding to regulate the activity of the SE where it is located).

Another interesting finding of this study is the dynamic change of the SE (surrounding rs10786700) activity in neurodevelopment. The activity of the SE containing rs10786700 is high (marked by strong EP300, DNase-Seq, H3K27ac and H3K4me1 signals) at early developmental stages (such as in embryonic stem cells and neural stem cells). Therefore, rs10786700 has a stronger regulation effect at these early developmental stages. However, the SE activity decreases gradually as development progresses. This finding revealed the complex and dynamic regulatory mechanism of the SCZ risk SNP rs10786700. This result also indicates that we need to pay special attention to the spatiotemporal effect of risk variants when investigating the regulatory mechanisms of SCZ risk variants.

The third interesting finding of this study is that rs10786700 regulates the expression of *SUFU*, which is a negative regulator of the hedgehog (HH) signalling pathway (Supplementary Fig. 1^1^). The HH signalling pathway is essential for normal embryonic development and human brain development.^94,95^ Mammals have three HH genes, including sonic hedgehog (*Shh*), Indian hedgehog (*Ihh*) and desert hedgehog (*Dhh*), and they are expressed in different tissues.^96^*Shh* (but not *Ihh* and *Dhh*) is widely expressed in human and mouse brain tissues (Supplementary Fig. 1^2^; data from The Human Protein Atlas, https://www.proteinatlas.org/). Lines of evidence suggest that the SHH signalling pathway plays a role in SCZ. First, the SHH pathway has been reported to be associated with SCZ.^97,98,99^ Second, antipsychotic drugs (haloperidol, clozapine and imipramine) inhibit the hedgehog signalling pathway by upregulating 7-dehydrocholesterol reductase,^100^ a sonic hedgehog signalling inhibitor.^101^ Third, the expression of *DISC1*, an SCZ risk gene, was altered in sonic hedgehog-mutant embryos.^102^ As a negative regulator of hedgehog signalling, SUFU is mainly located in the cytosol and nucleus, and is widely expressed in various tissues (Supplementary Fig. 1^3^; data from The Human Protein Atlas, https://www.proteinatlas.org/). *SUFU* has a crucial function in embryo development^103,104^ and the deletion of *Sufu* causes embryo lethality.^104^ Previous studies have revealed that *SUFU* is a tumour suppressor gene that inhibits the proliferation of cancer cells.^105,106^ In addition, *SUFU* also plays a vital role in cell differentiation. For example, *SUFU* deficiency delays cerebellar cell differentiation and *Sufu* deletion can inhibit proliferation of osteoprogenitor cells,^107^ ventral retinal progenitor cells and mNSCs.^108,109^ Our findings are consistent with these results (i.e. *Sufu* knockdown inhibits proliferation and neurogenesis of mNSCs). Of note, transcriptome data showed that *Sufu* knockdown affected the expression of SHH signalling pathway-related genes, including *Ptch1*, *Gli1* and *Gli3* (adjusted *P* < 0.05, Supplementary Table 7). Western blot also confirmed the effect of *Sufu* knockdown on PTCH1 expression (Fig. 5F and G). Our findings provide evidence that support the role of *SUFU* in SCZ (by regulating the SHH signalling pathway that affects neurodevelopment). As a matter of fact, neurodevelopmental hypothesis of SCZ has received much support.^2,3,4–110^ First, genes disrupted by rare structural variants in SCZ were enriched in neurodevelopmental pathways.^111^ Second, Gulsuner *et al*. showed that genes disrupted by *de novo* mutations in SCZ were mainly involved in a foetal prefrontal cortical network.^112^ Third, many SCZ risk genes, including *DISC1*,^52^*ZNF804A*,^113^*RELN*,^54^*C4A*,^114^*GLT8D1*,^34^*TMEM180*,^25^ etc. have been reported to be involved in neurodevelopment. These lines of evidence support the neurodevelopmental hypothesis of SCZ and suggest that rs10786700 may contribute to SCZ risk by regulating *SUFU*, a gene with an important role in neurodevelopment. Although many studies have shown the pivotal role of SHH signalling in SCZ, the genes in the SHH signalling pathway were not highlighted in recent SCZ GWASs. For the first time, our study links *SUFU* (a critical regulator of the SHH signalling pathway) to SCZ. Our study demonstrated how a risk variant reported by GWAS confers SCZ risk by regulating the SHH signalling pathway.

Several studies have demonstrated that SCZ associations were enriched in genes that are highly expressed in early brain developmental stage. For example, Cameron *et al.* recently conducted single nuclei RNA sequencing using five regions of the human prenatal brain, and they found significant enrichments of SCZ associations in genes with high expression level in developing populations.^115^ In addition, this study also showed that SCZ risk variants were significantly enriched in genes with important functions in neurodevelopment and synaptic signalling.^115^ In addition, Gulsuner *et al*. also found that perturbations of foetal prefrontal cortical neurogenesis have a critical role in the pathophysiology of schizophrenia.^112^ These findings are consistent with our results. We found that the schizophrenia risk variant rs10786700 regulates the expression of *SUFU* gene, which is highly expressed in the frontal cortex in early stage of development (Fig. 3H) and in excitatory neurons (Supplementary Fig. 9). In addition, *SUFU* affects neurodevelopment and dendritic spine morphogenesis.

Structural brain abnormalities have been widely reported in SCZ.^116,117^ We thus explored if rs10786700 is associated with brain volumetric phenotypes, using the data from Zhao *et al*.^42^ rs10786700 showed the most significant association with grey matter volume (Supplementary Table 8). In addition, significant associations between rs10786700 and other brain volumetric phenotypes were also observed, including the fourth ventricle, cerebellar vermal lobules and brainstem (Supplementary Table 8). We further examined if rs10786700 is associated with cognitive function using the data from Davies *et al*.^43^ No significant association was found between rs10786700 and cognition (Supplementary Table 9). These results suggest that rs10786700 may influence brain volumetric phenotypes by affecting neurodevelopment.

There are several limitations in this study. First, the presence of multiple binding bands in EMSA results suggests the complex regulatory effect of rs10786700 (Fig. 2I and J). However, we only focused on REST and EP300 in this study. Second, binding band 3 was significantly weakened after the addition of REST antibody or EP300 antibody (Fig. 2J, Fig. 3D), suggesting that REST and EP300 might form complexes to exert their regulatory effect. Third, we explored expression quantitative trait loci data generated using human brain tissues from prenatal and adulthood stages (Supplementary Fig. 1^4^).^26,27^ However, no significant correlation between rs10786700 and *SUFU* expression was observed. A possible reason is the relatively small sample size of the prenatal eQTL dataset. In addition, considering there are many cell types in the brain tissue, we could not detect association if the regulatory effect is cell type-specific. Finally, although our study revealed the potential function of *SUFU* in SCZ, the exact role and mechanisms of *SUFU* in SCZ remain to be elucidated.

In conclusion, our findings demonstrate that rs10786700 confers risk of SCZ by modulating the activity of the SE it located and regulating *SUFU* expression, a gene whose expression dysregulation plays a role in SCZ pathophysiology through affecting neurodevelopment and dendritic spine morphogenesis.

## Supplementary Material

awac352_Supplementary_Data
