# Supplementary material for: Genetic regulatory and biological implications of the 10q24.32 schizophrenia risk locus
Source: Brain. 2022 Sep 24;146(4):1403–19. doi: 10.1093/brain/awac352 (PMC10115178; doi:10.1093/brain/awac352)
Supplement: awac352_Supplementary_Data [file awac352_supplementary_data.pdf]

Supplementary materials and methods

Key resources table

Reagents, kits, software and instruments used in this study		
**Reagents and kits**	**Source**	**Identifier (Cat. No)**
pGL3-Promoter vector	Promega	E1761
pRL-TK	Promega	E2241
Lipofectamine™ 3000 transfection reagent	Invitrogen	L3000-015
Dual-Luciferase® Reporter 1000 Assay System	Promega	E1980
EMSA Probe Biotin Labeling Kit	Beyotime	GS008
Nuclear and Cytoplasmic Protein Extraction Kit	Beyotime	P0028
Chemiluminescent EMSA Kit	Beyotime	GS009
Nylon membrane	Millipore	INYC00010
pMD2.G	Addgene	12259
psPAX2	Addgene	12260
PEI	Polysciences	24765-1
Dulbecco's Modified Eagle Medium	Gibco	C11995500BT
Dulbecco's Modified Eagle Medium	Gibco	C12430500BT
DMEM/F12	Gibco	C11330500BT
Neurobasal™ Medium	Gibco	21103049
FBS	Gibco	10091148
Penicillin-Streptomycin	Gibco	15140-122
Sodium Pyruvate	Gibco	11360070
MEM Non-Essential Amino Acids Solution	Gibco	11140050
Laminin	Sigma	L2020-1mg
B-27 Supplement	ThermoFisher	12587010
N-2 Supplement	ThermoFisher	17502048
EGF	Stemcell	78006
bFGF	Stemcell	78003
Heparin Solution	Stemcell	07980
B-27 Supplement	ThermoFisher	17504044
GlutaMAX	Gibco	35050061
Lenti-X Concentrator	TaKaRa	631231
pSicoR-Ef1a-mCh-puro	Addgene	31845
Puromycin	Sigma	540222
TRIzol Reagent	Life technologies	15596018
PrimeScript RT reagent Kit with Gdna Eraser	TaKaRa	RR047B
TB Green Premix Ex Taq II(Tli RNaseH Plus)	TaKaRa	RR820B

Reagents and kits	Source	Identifier (Cat. No)
BrdU	Thermo	MA511282
Enhanced Cell Counting Kit-8	Beyotime	C0042
Immunol Staining Blocking Buffer	Beyotime	P0102
Anti-REST	Santa cruz	sc-374611
Anti-EP300	Santa cruz	sc-48343X
Anti-PAX6	Millipore	ab2237
Anti-NESTIN	Millipore	MAB353
Anti-SOX2	Santa Cruz	sc-17320
Anti-BrdU	Novus	NB500-235
Anti-GFAP	Sigma	G9269
Anti-MAP2	Millipore	AB5622
Anti-GFP	Abcam	ab13970
Anti-mCherry	GeneTex	GTX128508

Software	Source	Website
ClusterProfiler	Yu et al., 2012	http://bioconductor.org/packages/release/bioc/html/clusterProfiler.html
DESeq2	Love et al., 2014	http://www.bioconductor.org/packages/release/bioc/html/DESeq2.html
PLINK	Purcell et al., 2007	http://zzz.bwh.harvard.edu/plink/summary.shtml
NeuronStudio	Rodriguez et al., 2008	
GeneMapper	Applied Biosystems	

Instruments	Source
Mini chemi610 imaging system	Sagecreation
Spectrophotometer	BioTek
FluoViewTM FV1000 Laser Scanning Confocal Microscopy	Olympus
LSM 880 Basic Operation	Carl Zeiss
3730XL DNA analyzer	Applied Biosystems
Luminoskan Ascent chemiluminesence analyzer	Thermo scientific

Cell culture

HEK-293T, SH-SY5Y and SK-N-SH cell lines were purchased from the cell bank of Kunming institute of Zoology, Chinese Academy of Sciences, and cultured as previously described.[1] HEK293 cells were cultured in Dulbecco's Modified Eagle's medium (DMEM, Gibco, Cat. No: C11995500BT) containing 10% fetal bovine serum (FBS, Gibco, Cat. No: 10091148) and Penicillin-Streptomycin (100 units/mL) (Gibco, Cat. No: 15140-122). SK-N-SH and SH-SY5Y cells were cultured in DMEM (Gibco, Cat. No: C12430500BT, containing 10% FBS, 1 mM sodium pyruvate solution (Gibco, Cat. No: 11360070), 1×MEM non-essential amino acids solution (Gibco, Cat. No: 11140050), penicillin and streptomycin (100 units/mL). The mouse neural stem cells (mNSCs) were cultured in DMEM/F12 (1:1, Gibco, Cat. No: C11330500BT), 2% B-27 supplement (ThermoFisher, Cat. No: 12587010), 1% N-2 supplement

(ThermoFisher, Cat. No: 17502048), 20 ng/mL epidermal growth factor (EGF, Stemcell, Cat. No: 78006), 10 ng/mL basic fibroblast growth factor (bFGF, Stemcell, Cat. No: 78003), Penicillin-Streptomycin (100 units/mL), and 0.0002% heparin (20 μg/mL) (Stemcell, Cat. No: 07980), as previously described.[2-4] The differentiation medium consists of DMEM/F12, 2% B27 supplement, 1% N2 supplement, and 0.0002% heparin for mNSCs. The mNSCs cells were plated into cell culture plates (pre-coated with Laminin (Sigma, 20 μg/mL, Cat. No: L2020-1mg). The rat primary cortical neurons were cultured in neurobasal medium (Gibco, Cat. No: 21103049) containing 2% B27 supplement (ThermoFisher, Cat. No: 17504044) and 1 mM GlutaMAX supplement (Gibco, Cat. No: 35050061) as previously described.[5, 6] No mycoplasma contamination was found in this study.

Electrophoretic mobility shift assay (EMSA)

The reaction system of EMSA included 5 × EMSA/Gel-Shift Binding buffer (2 μL), nuclear extracts (1.5, 3.0, 4.5, 6.0 μg), biotin labeled probe (50 fmol) and nuclease-free water. For competitive assays, unlabeled probes (750 fmol) were added. For super-shift experiments, anti-REST antibody (Santa cruz, Cat. No: sc-374611, 0.8 μg) or anti-EP300 antibody (Santa cruz, Cat. No: sc-48343X, 1 μg, 1.5 μg) were added. In order to eliminate the nonspecific binding, the binding reaction mixtures were incubated for 10 min at 22 ℃ first. The biotin labeled probes were then added to the binding reaction mixtures and incubated for 20 minutes at 22 ℃. **S**amples were mixed with 10 × loading buffer and electrophoresed on 6.5% or 5% polyacrylamide gel at 100 V in 0.5 × TBE buffer for 70 min. The binding complexes were electrophoreticly transferred to Nylon membrane (Millipore, Cat. No: INYC00010) at 380 mA for 65 min or 100 min, and UV cross-linked for 15 min. The membrane was incubated with Streptavidin-HRP Conjugate after blocked with blocking buffer. Finally, the membrane was incubated with the substrate and pictures were collected using Mini chemi610 imaging system (SAGECREATION).

Knockdown experiments

Viruses packaging was carried out in HEK293T cells. 5 μg shRNA pSicoR-Ef1a-mCh-puro vector, 2.5 μg psPAX-2 (Addgene, Cat. No: 12259) and 1 μg PMD2. G (Addgene, Cat. No: 12260) were co-transferred into HEK293T cells (cultured in 6 cm dish) with PEI (vector/PEI = 1/2.5) (Polysciences,

Cat. No: 24765-1). Two days after transfection, the lentiviral particles were concentrated with Lenti-X Concentrator (TaKaRa, Cat. No: 631231) and were used to infect mNSCs (1/3 of the lentivirus particles). Two days after infection, the cells were selected with 2 μg/mL puromycin for 7 days. Knockdown efficiency of shRNAs was detected using quantitative real-time PCR (qPCR).

Genotyping

Genotyping were conducted with SNaPshot method as previously described.[7, 8] In brief, the 534 bp DNA fragments containing rs10786700 were amplified by PCR. The PCR products (containing rs1078670) were then used as template for single base extension, which was performed using ddNTPs labeled with different fluorescence. ABI 3730XL sequencer (Applied Biosystems, USA) was used to determine the genotype of the sample according to the color of the extended base, and the results were read out by GeneMapper software (Applied Biosystems, USA). We used Sanger sequencing to verify the results of genotyping. Samples failed to be genotyped with SNaPshot method were genotyped with Sanger sequencing.

Immunofluorescence staining

The mNSCs and rat primary cortical neurons were fixed with 4% Paraformaldehyde (PFA) and 4% PFA containing 4% sucrose for 15 min, respectively. And the cells were permeabilized by PBST for 15 min and blocked with blocking buffer (Beyotime, Cat. No: P0102) for 1 hour at room temperature. Primary antibodies were incubated at 4 °C for overnight and washed three times with PBS. Secondary antibodies were then incubated at room temperature for 1 hour in dark and washed three times. The mNSCs were counterstained with DAPI for 15 min, and photographed using FluoViewTM FV1000 Laser Scanning Confocal Microscopy (Olympus). The images of rat primary cortical neurons were captured LSM 880 Basic Operation (Carl Zeiss). The primary antibodies used in this study were: anti-BrdU (1:50, Novus, Cat. No: NB500-235), anti-PAX6 (Milipore, 1:500, Cat. No: ab2237), anti-NESTIN (Chemicon, 1:500, Cat. No: MAB353), anti-SOX2 (Santa cruze, 1:200, Cat. No: sc-17320), anti-GFAP (Sigma, 1:1000, Cat. No: G9269), anti-MAP2 (Millipore, 1:200, Cat. No: AB5622), Chicken polyclonal GFP antibody (Abcam, Cat. No: ab13970), and Rabbit polyclonal mCherry antibody (GeneTex, Cat. No: GTX128508).

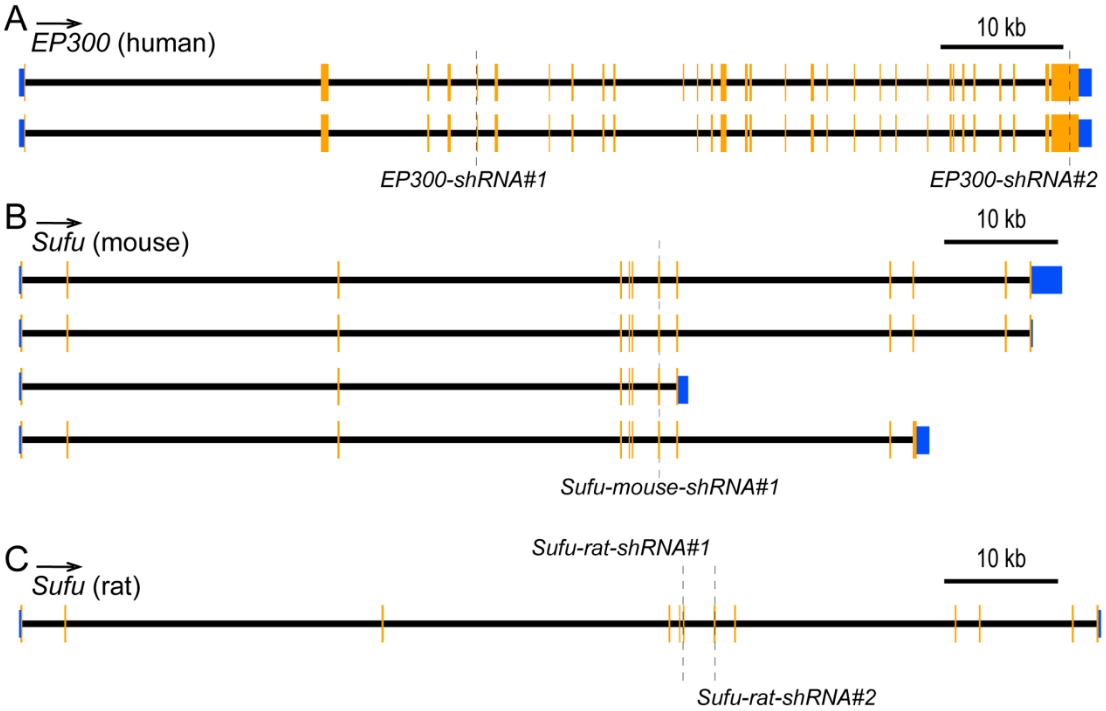

Supplementary Figure 1. The genomic regions targeted by the designed shRNAs. (**A**) The genomic regions targeted by the *EP300* shRNAs (human). (**B**) The genomic regions targeted by the *Sufu* shRNAs (mouse). (**C**) The regions targeted by the *Sufu* shRNAs (rat).

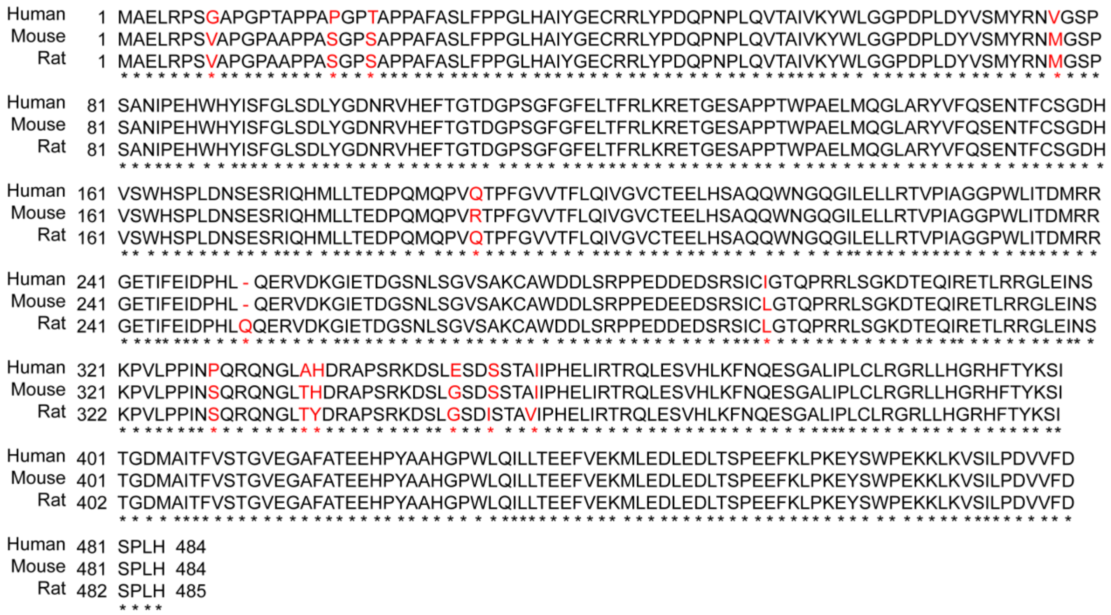

Supplementary Figure 2. Multiple alignments of SUFU protein sequences of human, mouse and rat were performed using COBALT.[9] Asterisks indicate evolutionarily conserved amino acid residues; red asterisk indicate the conservation across species of the histidine.

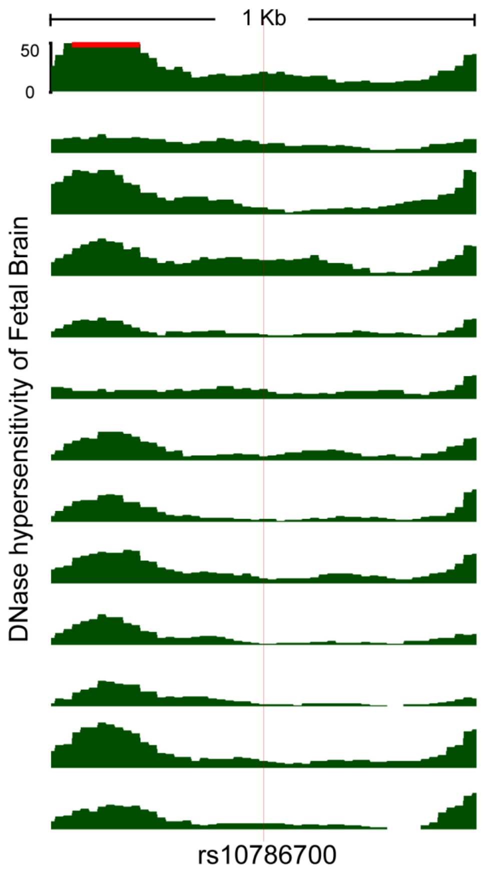

Supplementary Figure 3. The genomic region containing rs10786700 (1 kb) is an open chromatin region in human fetal brain. rs10786700 is located in an open chromatin region marked with strong DNase-Seq in human fetal brain.

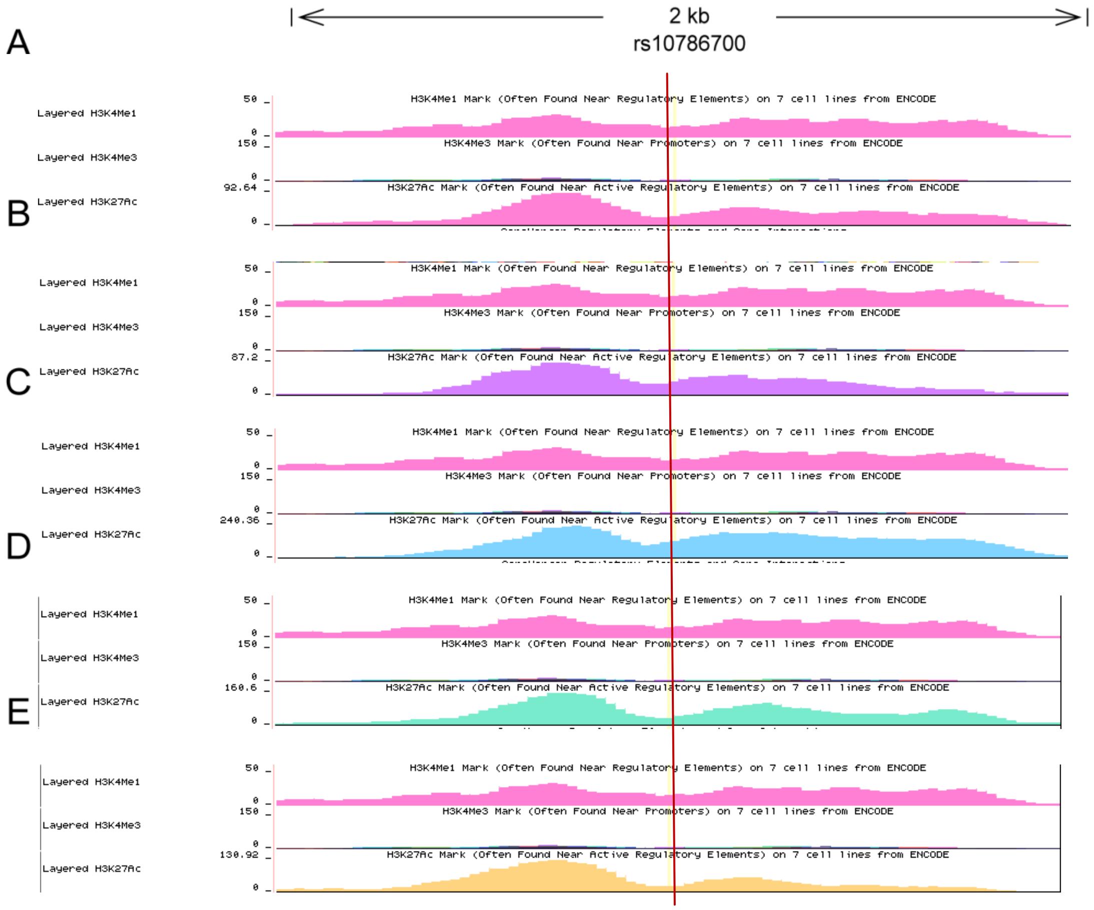

Supplementary Figure 4. The genomic region containing rs10786700 was also marked with H3K27ac signal in other cell lines. (**A-E**) The genomic region (2 kb centered on rs10786700) was also marked with H3K27ac signal in NHLF, NHEK, HUVEC, HSMM and H1-hESC cells. Data were from UCSC (http://genome.ucsc.edu/).

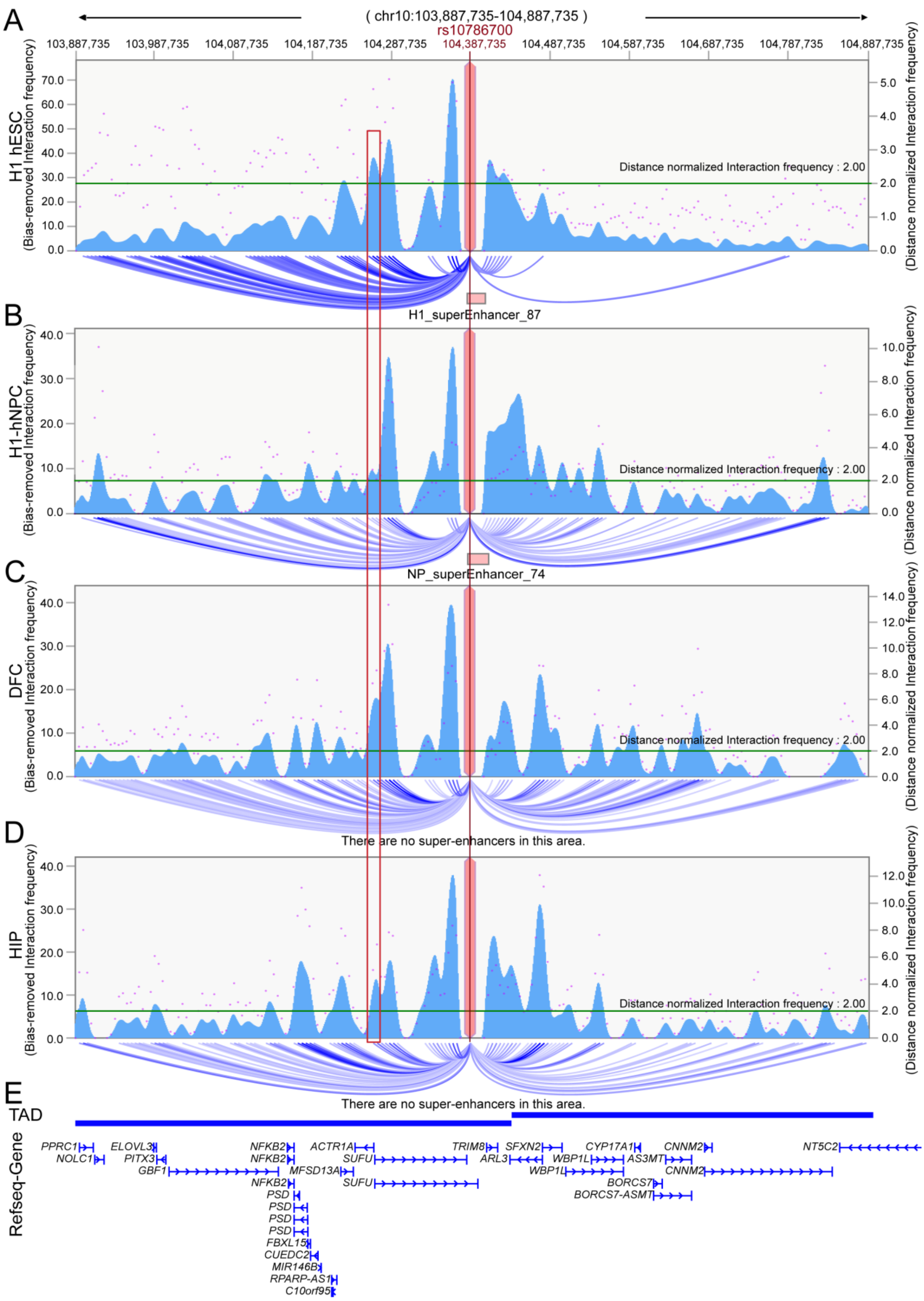

Supplementary Figure 5. Location and chromatin interaction patterns between rs10786700 and its nearby genes (1Mb region centered on rs10786700). (**A**) rs10786700 is located in a super enhancer (H1_superEnhancer_87) in H1 human embryonic stem cell (H1-hEST). (**B**) rs10786700 is located in a super enhancer (NP_superEnhancer_74) in H1-derived neuronal progenitor cell (H1-NPC). (**C**) In the dorsolateral prefrontal cortex (PFC), the genomic region containing rs10786700 was not marked with super enhancer signal. (**D**) In hippocampus, the genomic region containing rs10786700

was not marked with super enhancer signal. The y-axis values represent bias-removed interaction frequency, and distance normalized interaction frequency: 2. Data were from 3D Interaction Viewer and database (3DIV).[10] (**E**) rs10786700 and *SUFU* were located in the same topological associated domain (TAD) region (chr10:103,880,000-104,440,000).

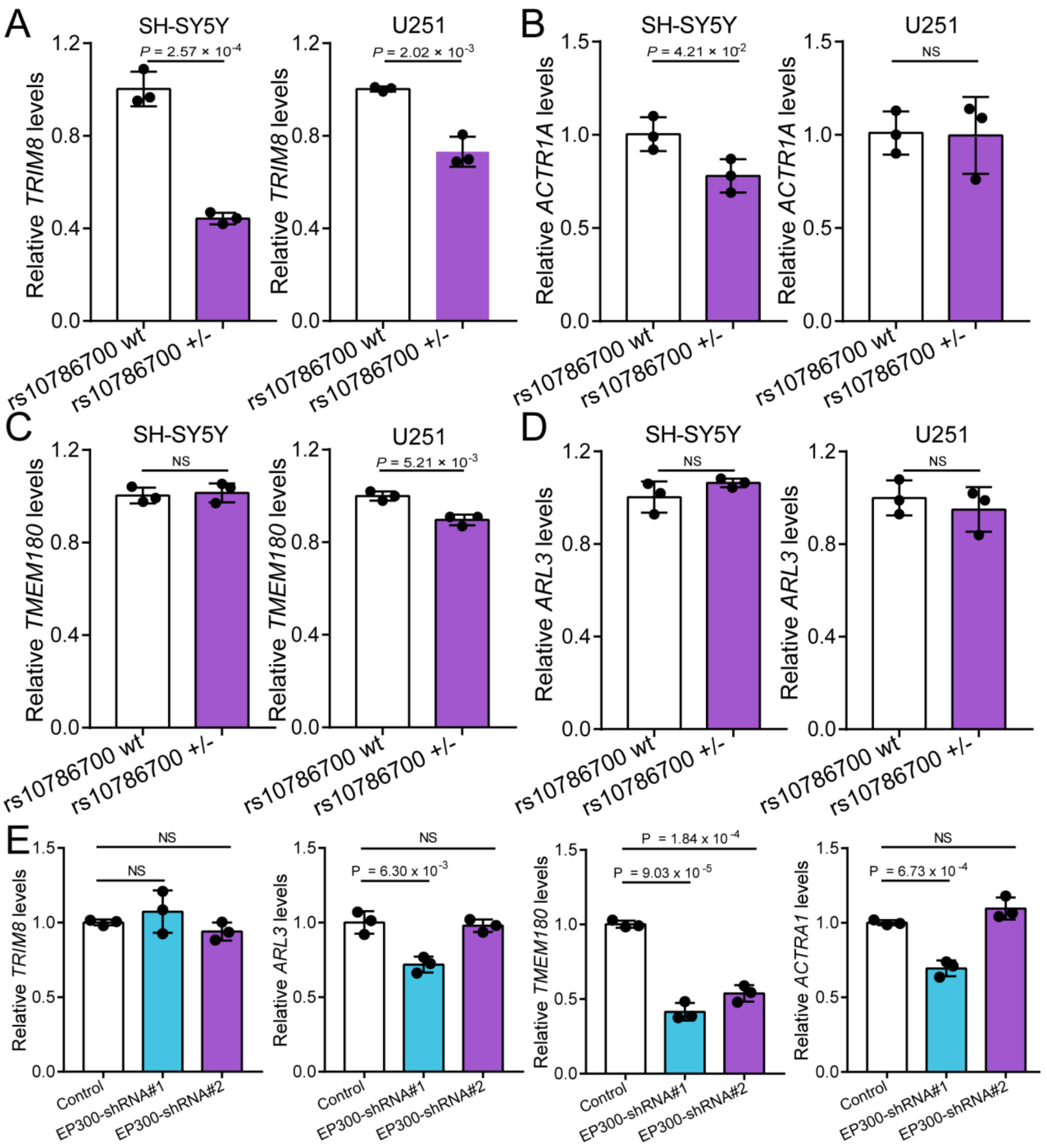

Supplementary Figure 6. The effect of rs10786700 deletion or *EP300* knockdown on the expression of the adjacent genes of rs10786700 in SH-SY5Y and U251 cells. (**A-D**) qPCR validation of *TRIM8*, *ACTR1A*, *TMEM180* and *ARL3* expression in rs10786700 knock-out SH-SY5Y and U251 cells. (**E**) qPCR validation of *TRIM8*, *ARL3*, *TMEM180* and *ACTR1A* expression in *EP300* knockdown SH-SY5Y cells. Unpaired two-tailed Student’s t-test; n = 3. Data are represented as mean ± SD. NS, not significant.

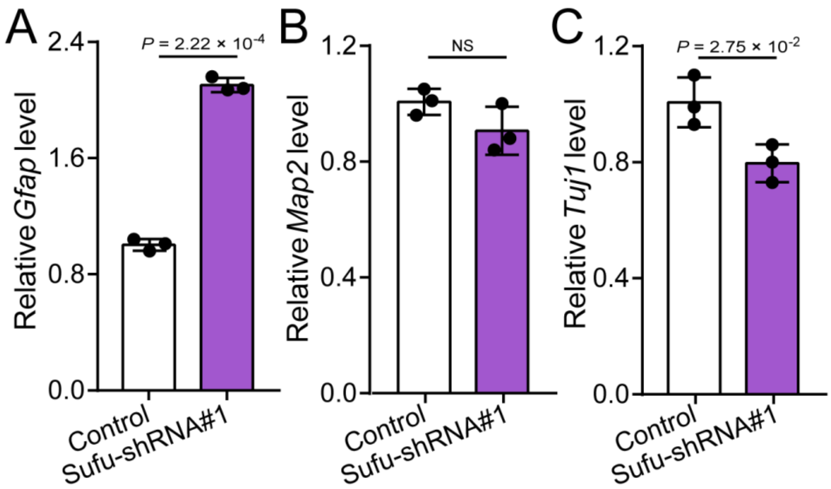

Supplementary Figure 7. qPCR validation of differentiation assays. (**A-C**) The results of qPCR for differentiation of mNSCs. Unpaired two-tailed Student's t-test; n = 3. Data are represented as mean ± SD. NS, not significant.

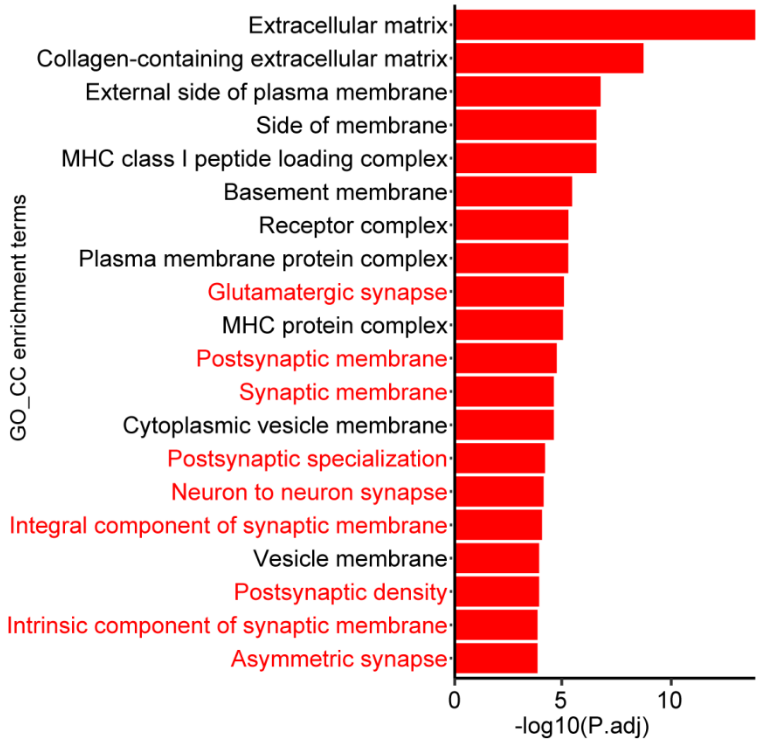

Supplementary Figure 8. GO (based on cell components, CC) analyses of 860 differentially expressed genes.

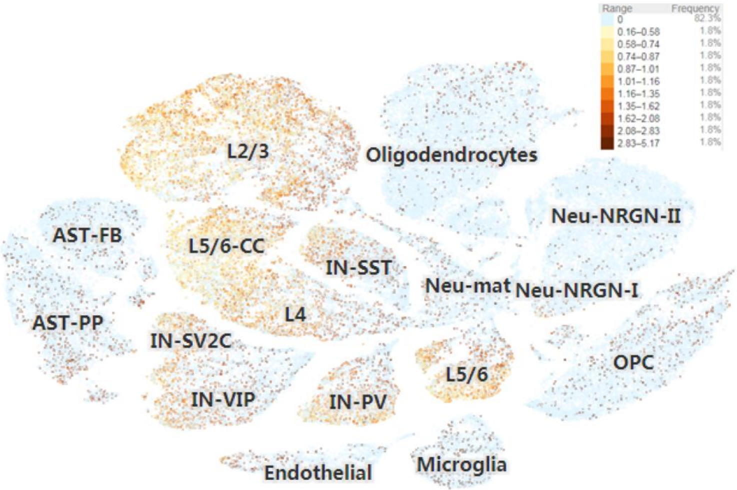

Supplementary Figure 9. *SUFU* expression in different cell types of the human brain. *SUFU* expression is relatively low in glial cells, such as astrocytes (AST-FB and AST-PP), microglia and oligodendrocytes (oligodendrocytes and OPC). However, *SUFU* is highly expressed in neurons, especially in excitatory neurons (L2/3, L4, L5/6 and L5/6-CC). Data were from UCSC cell Browser (https://autism.cells.ucsc.edu).[11]

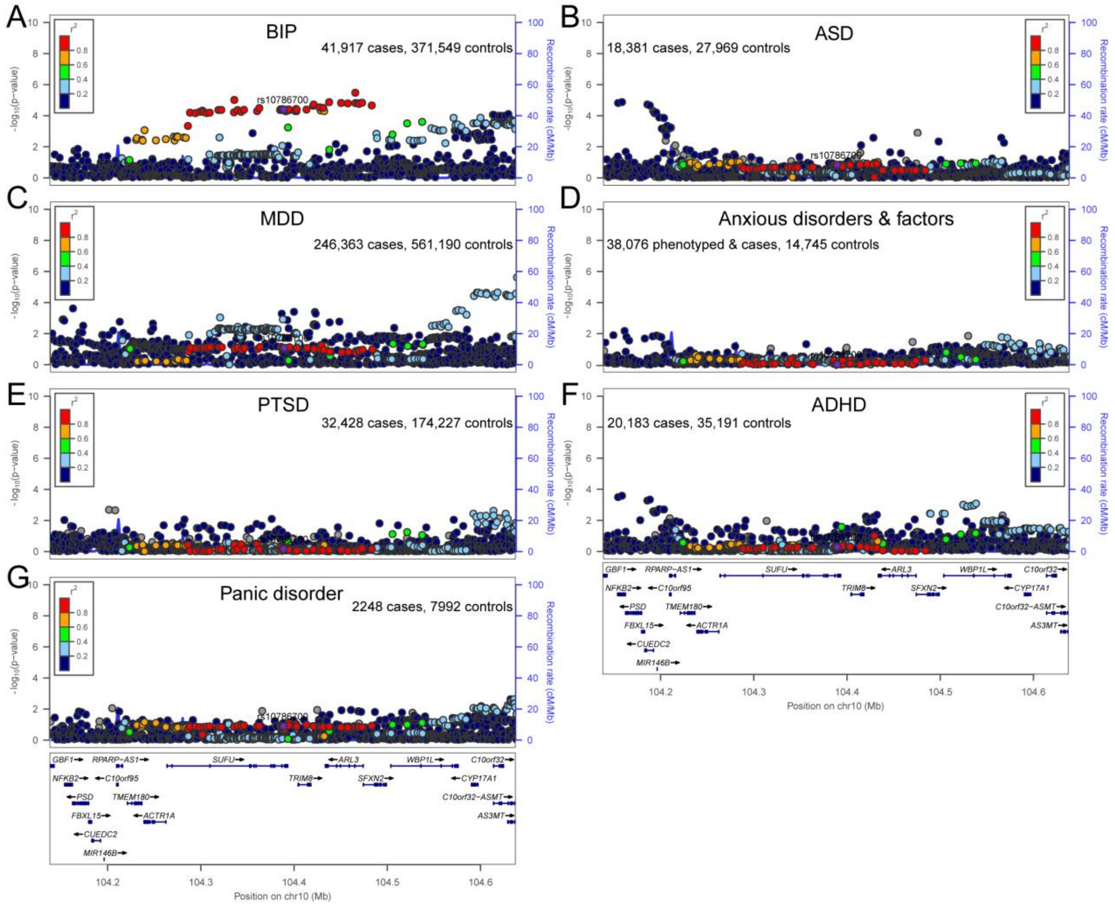

Supplementary Figure 10. The Locus zoom plots showing the associations between variants near rs10786700 (500 kb) and other psychiatric disorders. (**A-G**) Bipolar disorder (BIP), autism

spectrum disorder (ASD), major depressive disorder (MDD), anxious disorder, posttraumatic stress disorder (PTSD), attention deficit/hyperactivity disorder (ADHD) and panic disorder.

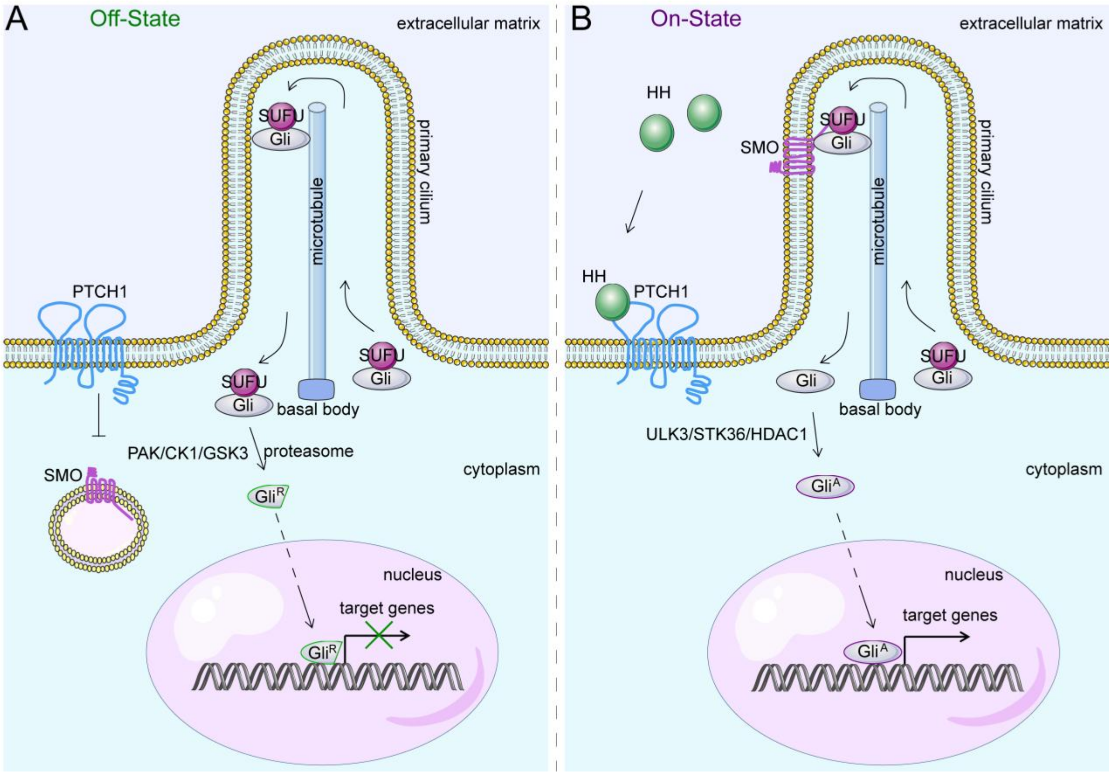

Supplementary Figure 11. The schematic diagram showing the role of SUFU in hedgehog (HH) pathway. (**A**) In the absence of HH, the receptor PTCH1 of HH prevents smoothened (SMO) from entering the cilia. SUFU forms a complex with GLI in the cytoplasm. GLI were phosphorylated by the kinase PAK/CK1/GSK3, and then hydrolyzed by the proteasome to form the transcriptional repressor GLI^R. GLI^R enters into the nucleus to inhibit the expression of target genes. (**B**) When the concentration of HH in the extracellular matrix increases, HH binds to the receptor PTCH1, lead to disinhibition of SMO, which enters the cilia and interacts with SUFU and GLI complex. GLI is then released from the complex and forms transcriptional activator GLI^A under the action of deacetylase HDAC1 or phosphorylase ULK3/STK36. GLI^A ectopically enters the nucleus to promote the expression of target genes.[12-15]

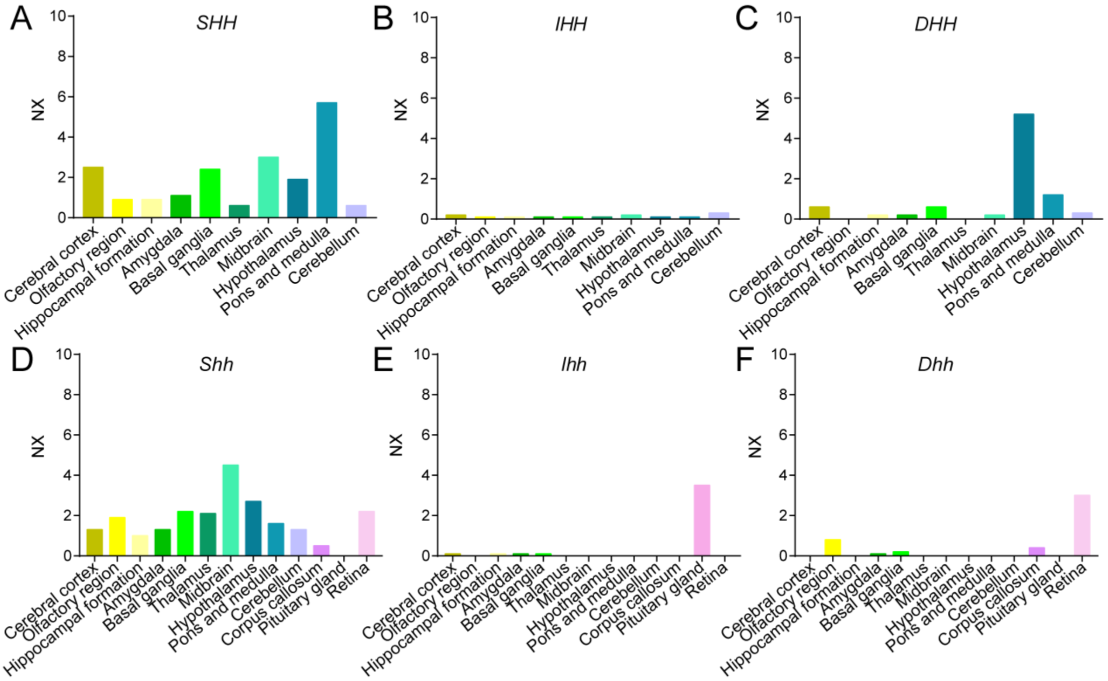

Supplementary Figure 12. The specific expression patterns of three hedgehog (HH) genes in human and mouse brain. (**A-C**) *SHH* (but not *IHH* and *DHH*) is widely expressed in human brain tissues. (**D-F**) *Shh* (but not *Ihh* and *Dhh*) is widely expressed in mouse brain tissues. (data were from The Human Protein Atlas, https://www.proteinatlas.org/) The y axis indicates normalized expression (NX) levels.

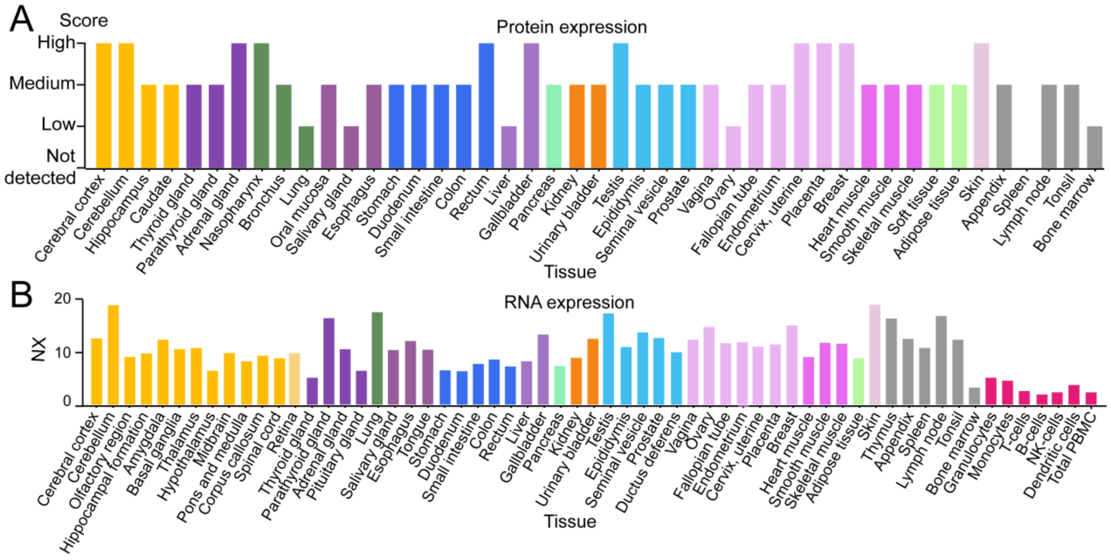

Supplementary Figure 13. The tissue-specific expression pattern of *SUFU*. (**A**) The tissue-specific protein expression pattern of *SUFU*. The y axis indicates expression levels. The x axis shows 45 tissue types. (**B**) The tissue-specific RNA expression pattern of *SUFU*. The y axis indicates normalized expression (NX) levels. The x axis shows 55 tissue types and 6 blood cell types. One color indicates one tissue group.

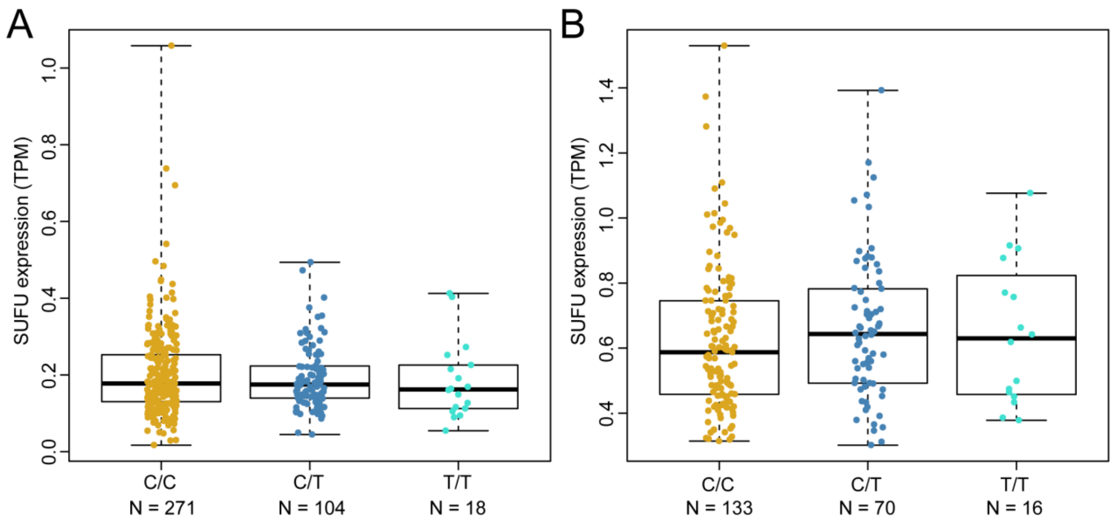

Supplementary Figure 14. eQTL analysis between rs2270363 and *SUFU* expression in the human brain. (**A**) The box-plot of eQTL results in adulthood human (expression data were from Collado-Torres *et al*).[16] (**B**) The box-plot of eQTL results in prenatal human brain (expression data were from Walker *et al*).[17]

Supplementary Table 1. Primers used for SNaPShot genotyping and dual-luciferase reporter gene assays.

Primers	Sequence (5' to 3')
rs10786700F	GCATCCTTATTTGCGAGGGCTT
rs10786700R	GGGTGCTTGATGGCTTTGGAAT
Ex-rs10786700 [a]	（TTTTT）2 GGATGGGAGCTTTCCTGGCC
rs10786700-WT-F[b]	CCAGAACATTTCTCTATCGATAGGTACCGAGGAATGGAGTCCATAATCCCCTT
rs10786700-WT-R[b]	GATGCAGATCGCAGATCTCGAGGGGTGCTTGATGGCTTTGGAAT
rs10786700-mut-F[c]	GTGAGACCTGGGAGCTGA GGCTCCC
rs10786700-mut-R[c]	GCTCCCAGGTCTCACCTT CTCAAGCGCT

[a]The extension primers were used for genotype.

[b]The primers were used to amplify the DNA fragments containing rs10786700.

[c]The primers were used for PCR-mediated site mutation.

Supplementary Table 2. The probes used for EMSA.

Probes	Sequence (5' to 3')
rs10786700-EMSA-CF	GGATGGGAGCTTTCCTGGCCCCAGTGCCCCATGGGTTAGTG
rs10786700-EMSA-CR	CACTAACCCATGGGGCACTGGGGCCAGGAAAGCTCCCATCC
rs10786700-EMSA-TF	GGATGGGAGCTTTCCTGGCCTCAGTGCCCCATGGGTTAGTG
rs10786700-EMSA-TR	CACTAACCCATGGGGCACTGAGGCCAGGAAAGCTCCCATCC

The base of rs10786700 was marked with red.

Supplementary Table 3. sgRNAs and shRNAs in this study.

sgRNAs or shRNAs	Sequence (5' to 3')
rs10786700-up-F	caccgCAGGAAAGCTCCCATCCCAC
rs10786700-up-R	aaacGTGGGATGGGAGCTTTCCTGc
rs10786700-down-F	caccgAGGGTCACCTCAATAGAGCA
rs10786700-down-R	aaacTGCTCTATTGAGGTGACCCTc
Sufu-shRNA#1-F	TGGCATTGAGACAGACGGTTTTCAAGAGAAACCGTCTGTCTCAATGCCTTTTTTC
Sufu-shRNA#1-R	TCGAGAAAAAAGGCATTGAGACAGACGGTTTCTCTTGAAAACCGTCTGTCTCAATGCCA
Sufu-rat-shRNA#1-F	TGAGATCGATCCACACCTGCTTCAAGAGAGCAGGTGTGGATCGATCTCTTTTTTC
Sufu-rat-shRNA#1-R	TCGAGAAAAAAGAGATCGATCCACACCTGCTCTCTTGAAGCAGGTGTGGATCGATCTCA
Sufu-rat-shRNA#2-F	TGCATCGAGACAGACGGTTCTTCAAGAGAGAACCGTCTGTCTCGATGCTTTTTTC
Sufu-rat-shRNA#2-R	TCGAGAAAAAAGCATCGAGACAGACGGTTCTCTCTTGAAGAACCGTCTGTCTCGATGCA

Supplementary Table 4. Primers used for real-time quantitative PCR

Primers	Sequence (5' to 3')
TMEM180-qPCR-F	GGAGCATCTGTTGTCCGACC
TMEM180-qPCR-R	GATGGGGAGCGACATAGGAGA
ACTRA1-qPCR-F	ATGCTGTGCCCATCTA
ACTRA1-qPCR-R	AGTCGTAGCCCTCCTT
SUFU-qPCR-F	AGACCCCTTGGACTATGTTAGC
SUFU-qPCR-R	CGAAGCTGATGTAGTGCCAGT
TRIM8-qPCR-F	ACAACGCCTACCGCCTCTA
TRIM8-qPCR-R	ATTTCATTCCTTCGGATCTCC
ALR3-qPCR-F	TGGGACATTGGTGGACAGAG
ALR3-qPCR-R	GTAATTCCGCTAGTTCCTGACC
Sufu-mouse&rat-qPCR-F2	ATGCTGCTGACGGAGGACC
Sufu-mouse&rat-qPCR-R2	CCACTGTTGGGCTGAATGTAA
ACTB-qPCR-F	CATGTACGTTGCTATCCAGGC
ACTB-qPCR-R	CTCCTTAATGTCACGCACGAT
Actb-mouse-qPCR-F	GGCTGTATTCCCCTCCATCG
Actb- mouse-qPCR-R	CCAGTTGGTAACAATGCCATGT
Actb-rat-qPCR-F	GGCCGTCTTCCCCTCCATCG
Actb- rat-qPCR-R	CCAGTTGGTGACAATGCCGTGT
Gfap-qPCR-F	CGGAGACGCATCACCTCTG
Gfap-qPCR-R	AGGGAGTGGAGGAGTCATTCG
Map2-qPCR-F	GCCAGCCTCAGAACAAACA
Map2-qPCR-R	GCTCAGCGAATGAGGAAGGA
Tuj1-qPCR-F	TAGACCCCAGCGGCAACTAT
Tuj1-qPCR-F	GTTCCAGGTTCCAAGTCCACC
Gpr17-qPCR-F	GACCTGTCCTGCGTGTTG
Gpr17-qPCR-R	TGTTCAGATAGAAGAGGAAGCC
Cbs-qPCR-F	GGAGAAGTGCCCTGGCTGTA
Cbs-qPCR-R	CCACCTCATAGGCTGTTTGCT
Col5a3-qPCR-F	CGGGGTACTCCTGGTCCTAC
Col5a3-qPCR-R	GCATCCCTACTTCCCCCTTG
Ppp2r2c-qPCR-F	GTTCCCTGTATGAGAGTGACTG
Ppp2r2c-qPCR-R	ATGCGGAAGAAGTTGTTGT
Anks1b-qPCR-F	ACTGGTGGCTCCCTAGA
Anks1b-qPCR-R	TTGGTCCAGATTTCACAG

Supplementary Table 5. The associations between 132 TF binding-disrupting SNPs and SCZ

SNP	GWAS_P	SNP	GWAS_P	SNP	GWAS_P	SNP	GWAS_P
rs3131340/rs115800871	1.57E-26	rs223390	9.70E-09	rs6992091	2.03E-06	rs494791	6.63E-05
rs10786700	9.76E-21	rs2027349	1.60E-08	rs3904682	2.70E-06	rs12114661	7.93E-05
rs12416331	1.89E-20	rs982085	2.12E-08	rs11160502	2.80E-06	rs340836	9.19E-05
rs281759	1.18E-17	rs117178087	2.44E-08	rs13323091	3.97E-06	rs2856268	0.0001081
rs796364	1.26E-17	rs4924832	2.48E-08	rs12665974	4.00E-06	rs13003881	0.0001405
rs3769481	1.40E-17	rs3813498	3.04E-08	rs7014953	4.13E-06	rs3773744	0.0001643
rs28594416	8.61E-14	rs2304206	3.32E-08	rs3794993	4.26E-06	rs3773745	0.0001995
rs60754073	9.99E-14	rs2304204	3.41E-08	rs2270363	4.59E-06	rs486781	0.0002816
rs4759413	1.40E-13	rs778593	3.46E-08	rs308699	4.85E-06	rs2385395	0.0002959
rs1805579	1.68E-13	rs4932217	3.90E-08	rs7304243	7.24E-06	rs10038801	0.0003131
rs76514049	1.83E-13	rs78681982	7.56E-08	rs11233566	1.04E-05	rs3096324	0.0003753
rs78866909	2.04E-13	rs6795127	1.70E-07	rs9616382	1.07E-05	rs12136320	0.0004923
rs34455584	5.06E-13	rs12146541	1.84E-07	rs9616378	1.10E-05	rs13113099	0.000499
rs12912934	9.47E-13	rs10860964	2.12E-07	rs41285977	1.14E-05	rs11263852	0.0005045
rs71196093/rs72748702	2.97E-12	rs1352318	2.20E-07	rs9985472	1.18E-05	rs2231318	0.0005751
rs10795	5.51E-12	rs34419497	2.31E-07	rs138833	1.23E-05	rs7662342	0.0006777
rs4790347	7.51E-12	rs1499894	2.42E-07	rs322004	1.82E-05	rs13193668	0.0009457
rs12473400	7.70E-12	rs78751650	3.13E-07	rs3804536	2.02E-05	rs78532287	0.001099
rs3814880	2.16E-11	rs58848914	3.50E-07	rs910800	2.32E-05	rs393223	0.001107
rs10083370	3.97E-11	rs1801311	3.68E-07	rs4786494	2.61E-05	rs7012106	0.001153
rs10083367	4.28E-11	rs28633410	3.77E-07	rs57336534	2.67E-05	rs9362397	0.001627
rs159961	6.35E-11	rs6002621	4.48E-07	rs2438345	2.74E-05	rs74105186	0.00184
rs3797040	8.64E-11	rs6871683	5.80E-07	rs790475	3.03E-05	rs9373388	0.001912
rs301792	9.63E-11	rs2269524	8.15E-07	rs16937	3.12E-05	rs321964	0.004759
rs3822346	2.45E-10	rs2974999	9.21E-07	rs1321	3.24E-05	rs2711116	0.005191
rs732381	9.37E-10	rs485315	9.76E-07	rs7410601	3.35E-05	rs7579996	0.005752
rs9611198	1.37E-09	rs11861362	1.01E-06	rs2551945	3.51E-05	rs11081564	0.01346
rs2535629	1.87E-09	rs9306356	1.05E-06	rs10940235	3.65E-05	rs74863866	0.02411
rs3743078	2.17E-09	rs1565231	1.14E-06	rs11986122	4.06E-05	rs61660810	0.04898
rs2675960	2.43E-09	rs223387	1.28E-06	rs11993089	4.85E-05	rs6919476	0.05211
rs72694957	3.63E-09	rs5751195	1.36E-06	rs3781884	5.47E-05	rs28365992	0.6921
rs10852932	6.53E-09	rs4785581	1.41E-06	rs61202914	5.63E-05	rs7796648	0.6928
rs716881	6.54E-09	rs8135801	1.81E-06	rs62021888	6.32E-05	rs2905232	0.8535

Supplementary Table 6. rs10786700 is located in super enhancer element in a variety of tissues and cells

SE_ID	SE_chr	SE_start	SE_end	Sample_ID	Tissue_type	Biosample_name	SE_rank
SE_01_06900090	chr10	104355660	104439361	Sample_01_069	Adrenal gland	adrenal-gland	90
SE_01_07000150	chr10	104355210	104437200	Sample_01_070	Ascending aorta	ascending-aorta	150
SE_02_07600351	chr10	104385900	104406736	Sample_02_076	Autonomic ganglia	SH-SY5Y_DMSO_3h	351
SE_02_34100295	chr10	104378707	104411187	Sample_02_341	Autonomic ganglia	SH-SY5Y_untreat	295
SE_02_30600234	chr10	104382875	104436674	Sample_02_306	Autonomic ganglia	SH-SY5Y	234
SE_02_25100143	chr10	104384152	104437842	Sample_02_251	Autonomic ganglia	SH-SY5Y-MYCN-OFF	143
SE_02_12600314	chr10	104370243	104442679	Sample_02_126	Blood	Induced-regulatory-T-cells_DMSO	314
SE_02_08100274	chr10	104356111	104436156	Sample_02_081	Blood	Monocyte-derived_dendritic_cells	274
SE_02_12700271	chr10	104370462	104442418	Sample_02_127	Blood	Induced-regulatory-T-cells_CPI-703	271
SE_00_00600123	chr10	104385031	104413416	Sample_00_006	Blood	CD14-positive-monocyte	123
SE_02_34400055	chr10	104358863	104412348	Sample_02_344	Blood	CD34	55
SE_02_34300138	chr10	104352840	104421567	Sample_02_343	Bone marrow	HSPC_A0	138
SE_02_06900215	chr10	104381805	104407227	Sample_02_069	Brain	SF268	215
SE_02_25300197	chr10	104374609	104410952	Sample_02_253	Brain	BE2C_DMSO	197
SE_02_42100172	chr10	104374675	104410584	Sample_02_421	Brain	BE2C_3	172
SE_02_34000132	chr10	104383537	104436360	Sample_02_340	Brain	BE2C_2	132
SE_02_15800069	chr10	104387117	104434689	Sample_02_158	Brain	U87_EGFRvIII_erlotinib	69
SE_02_22000067	chr10	104374971	104407784	Sample_02_220	Brain	BE2C_1	67
SE_02_41300060	chr10	104387219	104411555	Sample_02_413	Brain	GBM_2493	60
SE_01_07200067	chr10	104355477	104452101	Sample_01_072	Breast epithelium	breast-epithelium	67
SE_02_00500355	chr10	104386605	104407272	Sample_02_005	Cervical	cervical-cancer_KDM5C	355
SE_02_42700392	chr10	104387297	104407809	Sample_02_427	Colon	HCT116_shPAF1	392
SE_02_14300285	chr10	104387210	104406891	Sample_02_143	Colon	HCT116_Parental	285
SE_02_35200201	chr10	104355554	104407151	Sample_02_352	Colon	HCT116	201

SE_ID	SE_chr	SE_start	SE_end	Sample_ID	Tissue_type	Biosample_name	SE_rank
SE_02_05300013	chr10	104358620	104421039	Sample_02_053	Colon	DKO1	13
SE_02_11300093	chr10	104385925	104407142	Sample_02_113	Connective tissue	HT1080_DOX5d	93
SE_02_11200048	chr10	104352075	104407178	Sample_02_112	Connective tissue	HT1080_untreated	48
SE_01_07300127	chr10	104355572	104437766	Sample_01_073	Coronary artery	coronary-artery	127
SE_02_06500810	chr10	104386012	104411112	Sample_02_065	Embryo	KO2-Late_G1	810
SE_02_06400765	chr10	104386824	104409877	Sample_02_064	Embryo	DN-Early_G1	765
SE_02_12500732	chr10	104385971	104410305	Sample_02_125	Embryo	Embryonic-stem-cells_Primed	732
SE_02_03800563	chr10	104370697	104409821	Sample_02_038	Embryo	DE	563
SE_02_12400555	chr10	104387178	104418224	Sample_02_124	Embryo	Embryonic-stem-cells_Naive	555
SE_02_04000298	chr10	104385598	104410373	Sample_02_040	Embryo	PFG	298
SE_00_02400245	chr10	104386873	104406660	Sample_00_024	Embryo	mesendoderm	245
SE_02_03600229	chr10	104381280	104411934	Sample_02_036	Embryo	H7	229
SE_01_03300206	chr10	104386419	104409577	Sample_01_033	Embryo	H9	206
SE_02_01800206	chr10	104384778	104411116	Sample_02_018	Embryo	NEC	206
SE_00_01200189	chr10	104386441	104409787	Sample_00_012	Embryo	H1-hESC	189
SE_00_02300174	chr10	104385689	104407107	Sample_00_023	Embryo	mesenchymal-stem-cell	174
SE_02_01700129	chr10	104386790	104411992	Sample_02_017	Embryo	H9_ESC	129
SE_02_03900118	chr10	104384819	104391675	Sample_02_039	Embryo	AFG	118
SE_02_32800110	chr10	104363403	104393834	Sample_02_328	Embryo	H9_naive	110
SE_01_02300108	chr10	104355465	104406774	Sample_01_023	Embryo	cardiac-muscle-cell	108
SE_01_04700076	chr10	104385637	104411069	Sample_01_047	Embryo	mid-neurogenesis-radial-glial-cells	76
SE_01_05100057	chr10	104384801	104410666	Sample_01_051	Embryo	neural-progenitor-cell	57
SE_02_41600035	chr10	104386986	104390119	Sample_02_416	Embryo	H9_Het	35
SE_02_04500446	chr10	104386913	104406544	Sample_02_045	Embryo endodermal	CyT49_ES_D7	446
SE_02_04600373	chr10	104385364	104406733	Sample_02_046	Embryo endodermal	CyT49_ES_D10	373

SE_ID	SE_chr	SE_start	SE_end	Sample_ID	Tissue_type	Biosample_name	SE_rank
SE_02_04900240	chr10	104386970	104406186	Sample_02_049	Embryo endodermal	CyT49_GTtoH	240
SE_02_04800237	chr10	104386674	104406386	Sample_02_048	Embryo endodermal	CyT49_HE	237
SE_02_33000093	chr10	104374988	104439809	Sample_02_330	Endoderm	MHG	93
SE_02_25700338	chr10	104383992	104418679	Sample_02_257	Endometrioid	endometrioid_adenocarcinoma_TumorA	338
SE_02_27400017	chr10	104384192	104437276	Sample_02_274	Endometriosis	hTERT_KD	17
SE_02_10700146	chr10	104385769	104408501	Sample_02_107	Endometrium	EEC16	146
SE_01_07400167	chr10	104356255	104437053	Sample_01_074	Esophagus muscularis mucosa	esophagus-muscularis-mucosa	167
SE_01_07500070	chr10	104355595	104437415	Sample_01_075	Esophagus squamous epithelium	esophagus-squamous-epithelium	70
SE_02_10800275	chr10	104383834	104406880	Sample_02_108	Fallopian tube	FT246	275
SE_02_24400107	chr10	104375849	104439633	Sample_02_244	Foreskin	foreskin_D11	107
SE_00_01600077	chr10	104385945	104412000	Sample_00_016	Foreskin	iPS-DF-19.11	82
SE_02_41400066	chr10	104387118	104406213	Sample_02_414	Foreskin	BJEL	66
SE_01_07600477	chr10	104355769	104439178	Sample_01_076	Gastrocnemius medialis	gastrocnemius-medialis	477
SE_01_07700236	chr10	104355712	104436281	Sample_01_077	Gastroesophageal sphincter	gastroesophageal-sphincter	236
SE_02_33700308	chr10	104374836	104421487	Sample_02_337	Haematopoietic and lymphoid tissue	MV411_untreat	308
SE_02_38200598	chr10	104358625	104407747	Sample_02_382	Heart	purified cardiomyocyte G296S mutants	598
SE_02_38100326	chr10	104358210	104409903	Sample_02_381	Heart	purified cardiomyocyte WT	326
SE_00_01400281	chr10	104386155	104407663	Sample_00_014	Heart left ventricle	heart-left-ventricle	281
SE_01_07800147	chr10	104354771	104436135	Sample_01_078	Heart left ventricle	heart-left-ventricle	147
SE_02_18000753	chr10	104386996	104407216	Sample_02_180	Human melanoma	CJM	753
SE_02_18200220	chr10	104385770	104406737	Sample_02_182	Human melanoma	LOX-IMVI	220
SE_02_26800913	chr10	104386287	104407154	Sample_02_268	Kidney	A-498	913
SE_02_01100551	chr10	104353320	104406674	Sample_02_011	Kidney	A-498_wtVHL	551
SE_02_42900542	chr10	104386756	104406564	Sample_02_429	Kidney	12364284_VHL+	542
SE_02_14000509	chr10	104386634	104407090	Sample_02_140	Kidney	RT242	509

SE_ID	SE_chr	SE_start	SE_end	Sample_ID	Tissue_type	Biosample_name	SE_rank
SE_02_01200487	chr10	104386287	104406536	Sample_02_012	Kidney	786-O_non-targeting	487
SE_02_42800471	chr10	104386744	104407154	Sample_02_428	Kidney	12364284_VHL-	471
SE_02_40800377	chr10	104375866	104410365	Sample_02_408	Kidney	G401_Dox	377
SE_02_26700334	chr10	104385747	104406532	Sample_02_267	Kidney	786-O	334
SE_02_00800194	chr10	104386020	104406541	Sample_02_008	Kidney	786-O_empty_vector	194
SE_02_00900154	chr10	104385774	104406534	Sample_02_009	Kidney	786-O_wtVHL	154
SE_02_27000122	chr10	104385843	104406467	Sample_02_270	Kidney	PCS-400	122
SE_02_26900076	chr10	104385833	104391350	Sample_02_269	Kidney	HK-2	76
SE_00_02000746	chr10	104355698	104407164	Sample_00_020	Large intestine	large-intestine_108d	746
SE_02_32601070	chr10	104386569	104406854	Sample_02_326	Liver	hepatocytes_d3	1070
SE_02_28600497	chr10	104386131	104406760	Sample_02_286	Liver	HuH7_IL-1alpha_1h	497
SE_02_01500351	chr10	104386981	104406631	Sample_02_015	Liver	HepG2_shNS	351
SE_02_41200243	chr10	104386921	104439744	Sample_02_412	Liver	TTC549_Doxycycline	243
SE_01_03700165	chr10	104386338	104406990	Sample_01_037	Liver	hepatocyte	165
SE_02_34200074	chr10	104355008	104435958	Sample_02_342	Liver	HSPCs_F0	74
SE_02_17200508	chr10	104338821	104439294	Sample_02_172	Lung	IMR-90_quiescent	508
SE_02_21000403	chr10	104354853	104406466	Sample_02_210	Lung	MRC-5_N_1DPT	403
SE_02_20900376	chr10	104352654	104409850	Sample_02_209	Lung	MRC-5_MRC5_1DPT	376
SE_02_17500280	chr10	104386911	104435948	Sample_02_175	Lung	IMR-90_Etoposide	280
SE_01_02900240	chr10	104386348	104406919	Sample_01_029	Lung	fibroblast-of-lung	240
SE_00_02100167	chr10	104379049	104409854	Sample_00_021	Lung	lung_3y	167
SE_00_01500102	chr10	104379949	104410248	Sample_00_015	Lung	IMR-90	102
SE_02_16500081	chr10	104352413	104407080	Sample_02_165	Lung	H2087_LCC2	81
SE_02_21100067	chr10	104354793	104439386	Sample_02_211	Lung	MRC-5_NFD_1DPT	67
SE_02_05400059	chr10	104339501	104407527	Sample_02_054	Lung	IMR-90_DMSO_1	59

SE_ID	SE_chr	SE_start	SE_end	Sample_ID	Tissue_type	Biosample_name	SE_rank
SE_02_28200056	chr10	104380155	104438180	Sample_02_282	Lung	IMR-90	56
SE_02_05500051	chr10	104339580	104408738	Sample_02_055	Lung	IMR-90_Nutlin-3a	51
SE_02_16400051	chr10	104352413	104407080	Sample_02_164	Lung	H2087_Parental	51
SE_02_05700698	chr10	104380665	104406326	Sample_02_057	Mammary Gland	vHMEC	698
SE_02_16700673	chr10	104387561	104407001	Sample_02_167	Mammary Gland	HCC1954_LCC2	673
SE_02_15000462	chr10	104386925	104407130	Sample_02_150	Mammary Gland	MDA-MB-231_GATA3	462
SE_02_28000400	chr10	104386834	104407192	Sample_02_280	Mammary Gland	SUM159_DMSO_24h	400
SE_02_11400380	chr10	104386552	104411550	Sample_02_114	Mammary Gland	CAL51	380
SE_02_39900356	chr10	104386918	104406841	Sample_02_399	Mammary Gland	SUM159PT-BT-474	356
SE_02_09600345	chr10	104379832	104418957	Sample_02_096	Mammary Gland	ZR-75-1_1	345
SE_02_11600313	chr10	104385368	104395575	Sample_02_116	Mammary Gland	MDA-MB-231_untreat	313
SE_02_39500303	chr10	104385484	104406953	Sample_02_395	Mammary Gland	BT-549	303
SE_02_39700287	chr10	104387061	104411229	Sample_02_397	Mammary Gland	CAL51-MCF-7	287
SE_02_40300284	chr10	104381050	104419290	Sample_02_403	Mammary Gland	T-47D_untreat	284
SE_02_40500265	chr10	104387062	104407058	Sample_02_405	Mammary Gland	ZR-75-30_KO	265
SE_02_40200256	chr10	104386439	104406912	Sample_02_402	Mammary Gland	SUM159PT-ZR-75-1	256
SE_02_40100166	chr10	104384562	104407602	Sample_02_401	Mammary Gland	SUM159PT-T47D	166
SE_02_40600164	chr10	104387660	104406764	Sample_02_406	Mammary Gland	ZR-75-30_parental	164
SE_02_39800113	chr10	104385689	104407390	Sample_02_398	Mammary Gland	MDA-MB-231-MCF-7	113
SE_02_05600110	chr10	104378949	104439409	Sample_02_056	Mammary Gland	HMEC	110
SE_02_15100089	chr10	104385878	104408717	Sample_02_151	Mammary Gland	MDA-MB-231_TA1del	89
SE_00_02600151	chr10	104374929	104406970	Sample_00_026	Muscle of trunk	muscle-of-trunk	151
SE_02_30100756	chr10	104379341	104410011	Sample_02_301	Neuroblastoma	GIMEN	756
SE_02_29600539	chr10	104378706	104408226	Sample_02_296	Neuroblastoma	CLB-BER-Lud	539
SE_02_29700514	chr10	104382926	104411802	Sample_02_297	Neuroblastoma	CLB-CAR	514

SE_ID	SE_chr	SE_start	SE_end	Sample_ID	Tissue_type	Biosample_name	SE_rank
SE_02_30400489	chr10	104379545	104411508	Sample_02_304	Neuroblastoma	N206	489
SE_02_31600486	chr10	104380837	104407850	Sample_02_316	Neuroblastoma	CHP212	486
SE_02_31000450	chr10	104379172	104409930	Sample_02_310	Neuroblastoma	SJNB8	450
SE_02_31700345	chr10	104379835	104407528	Sample_02_317	Neuroblastoma	GICAN	345
SE_02_31500341	chr10	104379001	104406593	Sample_02_315	Neuroblastoma	TR14	341
SE_02_31100232	chr10	104374921	104439431	Sample_02_311	Neuroblastoma	SK-N-AS	232
SE_02_31300186	chr10	104379048	104439749	Sample_02_313	Neuroblastoma	SK-N-DZ	186
SE_02_31200137	chr10	104352647	104439890	Sample_02_312	Neuroblastoma	SK-N-BE_2-C	137
SE_02_30500112	chr10	104352594	104407141	Sample_02_305	Neuroblastoma	SH-EP	112
SE_02_33900064	chr10	104375153	104410343	Sample_02_339	Neuroblastoma	NGP	64
SE_02_26200323	chr10	104386139	104410358	Sample_02_262	Osteoblastic	hFOB_JQ1_differentiated	323
SE_02_26100257	chr10	104385234	104410427	Sample_02_261	Osteoblastic	hFOB_differentiated	257
SE_02_26300218	chr10	104385979	104410748	Sample_02_263	Osteoblastic	hFOB_undifferentiated	218
SE_02_07300757	chr10	104386195	104406764	Sample_02_073	Other	90-8TL_DMSO	757
SE_02_11000138	chr10	104338467	104407761	Sample_02_110	Ovaries	IOE11	138
SE_02_11100085	chr10	104385935	104407963	Sample_02_111	Ovaries	IOE4	85
SE_02_08600515	chr10	104386665	104439310	Sample_02_086	Pancreas	CAPAN1	515
SE_02_41900080	chr10	104379304	104439393	Sample_02_419	Pectoralis major muscle	LHCN-M2_nodox	80
SE_02_28400365	chr10	104371258	104437213	Sample_02_284	Pelvis	patient 119	365
SE_02_10300549	chr10	104387080	104413892	Sample_02_103	Peripheral blood	Macrophages_GM-CSF_72h_TNF-a_PGE2_Pam3CSK4	549
SE_02_10100471	chr10	104386990	104437980	Sample_02_101	Peripheral blood	Macrophages_GM-CSF_72h_IFN-y	471
SE_02_25600176	chr10	104374768	104421988	Sample_02_256	Peripheral blood	MV411	176
SE_02_17600089	chr10	104352836	104439481	Sample_02_176	Peripheral blood	LOUCY	89
SE_01_07900116	chr10	104356067	104437283	Sample_01_079	Peyers patch	Peyers-patch	116
SE_00_03101103	chr10	104386609	104407146	Sample_00_031	Placenta	placenta	1103

SE_ID	SE_chr	SE_start	SE_end	Sample_ID	Tissue_type	Biosample_name	SE_rank
SE_01_02700718	chr10	104384429	104418674	Sample_01_027	Prostate	epithelial-cell-of-prostate	718
SE_01_08000227	chr10	104380268	104419091	Sample_01_080	Prostate	prostate	227
SE_00_03200196	chr10	104359446	104407383	Sample_00_032	Psoas muscle	psoas-muscle_30y	196
SE_00_03300128	chr10	104374697	104412092	Sample_00_033	Psoas muscle	psoas-muscle_3y	128
SE_02_27700092	chr10	104384723	104412408	Sample_02_277	Retina	FW18	92
SE_02_27800060	chr10	104385354	104439921	Sample_02_278	Retina	FW24	60
SE_02_27500051	chr10	104378561	104440878	Sample_02_275	Retina	FW14	51
SE_02_27600033	chr10	104386473	104439934	Sample_02_276	Retina	FW15	33
SE_01_08100138	chr10	104356464	104439113	Sample_01_081	Right atrium auricular region	right-atrium-auricular-region	138
SE_01_08200162	chr10	104355121	104439308	Sample_01_082	Right lobe of liver	right-lobe-of-liver	162
SE_01_08300208	chr10	104356292	104437236	Sample_01_083	Sigmoid colon	sigmoid-colon	208
SE_02_36800357	chr10	104358753	104410384	Sample_02_368	Skeletal muscle	NS134	357
SE_02_26400297	chr10	104386542	104408748	Sample_02_264	Skeletal muscle	7250-empty	297
SE_02_36700266	chr10	104355994	104434903	Sample_02_367	Skeletal muscle	NS129	266
SE_02_36400236	chr10	104378742	104440185	Sample_02_364	Skeletal muscle	CTR	236
SE_02_37300113	chr10	104386908	104439546	Sample_02_373	Skeletal muscle	RMS206	113
SE_02_26500096	chr10	104379431	104439622	Sample_02_265	Skeletal muscle	7250-PAX3-FOXO1	96
SE_02_37200082	chr10	104387012	104439405	Sample_02_372	Skeletal muscle	RMS008	82
SE_02_36600076	chr10	104361060	104439603	Sample_02_366	Skeletal muscle	NCI0082	76
SE_02_38000070	chr10	104352297	104439814	Sample_02_380	Skeletal muscle	RH18	70
SE_02_37000069	chr10	104379235	104439620	Sample_02_370	Skeletal muscle	RH4	69
SE_02_37800042	chr10	104386912	104439952	Sample_02_378	Skeletal muscle	SCMC	42
SE_02_36900040	chr10	104385798	104439845	Sample_02_369	Skeletal muscle	RD	40
SE_02_37900040	chr10	104385798	104439845	Sample_02_379	Skeletal muscle	RD-Fusion-Negative-Rhabdomyosarcoma	40
SE_02_37100023	chr10	104379015	104439753	Sample_02_371	Skeletal muscle	RH5	23

SE_ID	SE_chr	SE_start	SE_end	Sample_ID	Tissue_type	Biosample_name	SE_rank
SE_02_42300423	chr10	104386714	104411322	Sample_02_423	Skin	A375	423
SE_02_06800311	chr10	104386965	104407400	Sample_02_068	Skin	A375_DMSO	311
SE_02_21600144	chr10	104377155	104418720	Sample_02_216	Skin	NHEK_scramble	144
SE_02_10600118	chr10	104378836	104405626	Sample_02_106	Skin	NHEK_M	118
SE_02_39600113	chr10	104352990	104439162	Sample_02_396	Skin	NHEK-D	113
SE_00_03600454	chr10	104358602	104409986	Sample_00_036	Small intestine	small-intestine_108days	454
SE_01_06600286	chr10	104380600	104407048	Sample_01_066	Smooth muscle	smooth-muscle-cell	286
SE_00_03800586	chr10	104384676	104394135	Sample_00_038	Spinal cord	spinal-cord_108d	586
SE_01_08400083	chr10	104338634	104439035	Sample_01_084	Spleen	spleen	83
SE_00_04300722	chr10	104385300	104395826	Sample_00_043	Stomach	stomach_96d	722
SE_01_08500055	chr10	104355488	104439491	Sample_01_085	Stomach	stomach	55
SE_01_08600240	chr10	104354654	104412523	Sample_01_086	Thoracic aorta	thoracic-aorta	240
SE_01_08700104	chr10	104386818	104449219	Sample_01_087	Thyroid gland	thyroid-gland	104
SE_01_08800101	chr10	104378455	104439840	Sample_01_088	Tibial nerve	tibial-nerve	101
SE_02_23400260	chr10	104367890	104443452	Sample_02_234	Tonsil	Th17	260
SE_02_23300227	chr10	104370006	104436550	Sample_02_233	Tonsil	Th1	227
SE_01_08900096	chr10	104354896	104437419	Sample_01_089	Transverse colon	transverse-colon	96
SE_02_08900398	chr10	104387026	104406756	Sample_02_089	Umbilical cord	umbilical-cord-derived-mesenchymal-stem-cells_SGA_cycle_3	398
SE_02_08800346	chr10	104386543	104406501	Sample_02_088	Umbilical cord	umbilical-cord-derived-mesenchymal-stem-cells_SGA_cycle_0	346
SE_02_09200201	chr10	104387008	104406604	Sample_02_092	Umbilical cord	umbilical-cord-derived-mesenchymal-stem-cells_Control_cycle_3	201
SE_02_09100150	chr10	104386606	104406793	Sample_02_091	Umbilical cord	umbilical-cord-derived-mesenchymal-stem-cells_Control_cycle_0	150
SE_02_09300137	chr10	104386993	104406737	Sample_02_093	Umbilical cord	umbilical-cord-derived-mesenchymal-stem-cells_Control_cycle_6	137
SE_02_23000118	chr10	104387111	104402518	Sample_02_230	Umbilical cord	ID60	118
SE_02_22900103	chr10	104387249	104403331	Sample_02_229	Umbilical cord	ID01	103
SE_02_02700086	chr10	104355236	104418542	Sample_02_027	Umbilical vein	HUVEC_VEGF_4h	86

SE_ID	SE_chr	SE_start	SE_end	Sample_ID	Tissue_type	Biosample_name	SE_rank
SE_01_09000014	chr10	104338486	104439524	Sample_01_090	Upper lobe of left lung	upper-lobe-of-left-lung	14
SE_02_17900201	chr10	104384936	104411564	Sample_02_179	Urinary bladder	T24	201
SE_01_09100187	chr10	104361029	104418771	Sample_01_091	Uterus	uterus	187
SE_01_09200068	chr10	104355647	104439641	Sample_01_092	Vagina	vagina	68

SE, super-enhancer. Data were from the comprehensive human Super-Enhancer database (SEdb) [18], http://www.licpathway.net/sedb/index.php.

Supplementary Table 7. Sufu knockdown affected the expression of SHH signaling pathway-related genes

gene	log2FoldChange	*P* value	*P* adj
Shh	-0.51	0.1135	0.3009
Ptch1	0.25	7.10×10^{-6}	8.35×10^{-5}
Ptch2	-0.30	0.5402	0.7709
Sufu	-0.82	3.10×10^{-24}	3.10×10^{-22}
Gli1	-2.08	0.0043	0.0239
Gli2	0.29	0.0494	0.1659
Gli3	0.55	0.0009	0.0059

Supplementary Table 8. Association between rs10786700 and regional brain volumes

Category	Phenotype	*P* value
sMRI	Grey matter	3.0×10^{-7}
sMRI	Ventricle_4th_pc1	3.4×10^{-7}
sMRI	Cerebellar_vermal_lobules_VIII.X_pc2	7.0×10^{-7}
sMRI	Cerebellar_vermal_lobules_VIII.X_pc4	1.2×10^{-6}
sMRI	Grey matter (pc2)	1.4×10^{-6}
sMRI	X4th.ventricle	2.5×10^{-6}
sMRI	Brain stem (pc5)	7.7×10^{-6}

Supplementary Table 9. Association between rs10786700 and cognition

rs10786700	Allele 1	Allele 2	Beta	*P* value
Memory	T	C	0.0044	0.57
Verbal–numerical reasoning	T	C	-0.0150	0.27
Reaction time	T	C	-0.0042	0.59

Beta is based on Allele 1.

References

1. Huo YX, Li SW, Liu JW, Li XY, Luo XJ. Functional genomics reveal gene regulatory mechanisms underlying schizophrenia risk. *Nat Commun* 2019; **10:** 670.
2. Louis SA, Mak CK, Reynolds BA. Methods to culture, differentiate, and characterize neural stem cells from the adult and embryonic mouse central nervous system. *Methods in molecular biology* 2013; **946:** 479-506.
3. Li JY, Liu J, Manaph NPA, Bobrovskaya L, Zhou XF. ProBDNF inhibits proliferation, migration and differentiation of mouse neural stem cells. *Brain research* 2017; **1668:** 46-55.
4. Wang JY, Li XY, Li HJ, Liu JW, Yao YG, Li M *et al.* Integrative analyses followed by functional characterization reveal TMEM180 as a schizophrenia risk gene. *Schizophrenia bulletin* 2021; DOI: 10.1093/schbul/sbab1032.
5. Cai X, Yang ZH, Li HJ, Xiao X, Li M, Chang H. A Human-specific schizophrenia risk tandem repeat affects alternative splicing of a human-unique isoform AS3MTd2d3 and mushroom dendritic spine density. *Schizophrenia bulletin* 2021; **47**(1)**:** 219-227.
6. Li S, Li J, Liu J, Wang J, Li X, Huo Y *et al.* Regulatory variants at 2q33.1 confer schizophrenia risk by modulating distal gene TYW5 expression. *Brain* 2021; DOI: 10.1093/brain/awab1357.
7. Li K, Li Y, Wang J, Huo Y, Huang D, Li S *et al.* A functional missense variant in ITIH3 affects protein expression and neurodevelopment and confers schizophrenia risk in the Han Chinese population. *Journal of genetics and genomics = Yi chuan xue bao* 2020; **47**(5)**:** 233-248.
8. Li YF, Ma CG, Li WQ, Yang YF, Li XY, Liu JW *et al.* A missense variant in NDUFA6 confers schizophrenia risk by affecting YY1 binding and NAGA expression. *Mol Psychiatr* 2021; DOI: 10.1038/s41380-41021-01125-x.
9. Sherman BT, Hao M, Qiu J, Jiao XL, Baseler MW, Lane HC *et al.* DAVID: a web server for functional enrichment analysis and functional annotation of gene lists (2021 update). *Nucleic Acids Res* 2022; **50**(W1)**:** W216-W221.
10. Yang DC, Jang I, Choi J, Kim MS, Lee AJ, Kim H *et al.* 3DIV: A 3D-genome Interaction Viewer and database. *Nucleic Acids Res* 2018; **46**(D1)**:** D52-D57.
11. Velmeshev D, Schirmer L, Jung D, Haeussler M, Perez Y, Mayer S *et al.* Single-cell genomics identifies cell type-specific molecular changes in autism. *Science* 2019; **364**(6441)**:** 685-689.
12. Taipale J, Cooper MK, Maiti T, Beachy PA. Patched acts catalytically to suppress the activity of Smoothened. *Nature* 2002; **418**(6900)**:** 892-897.
13. Hui CC, Angers S. Gli Proteins in Development and Disease. *Annu Rev Cell Dev Bi* 2011; **27:** 513-537.
14. Doheny D, Manore SG, Wong GL, Lo HW. Hedgehog Signaling and Truncated GLI1 in Cancer. *Cells-Basel* 2020; **9**(9).
15. Han YH, Wang B, Cho YS, Zhu J, Wu J, Chen YB *et al.* Phosphorylation of Ci/Gli by Fused Family Kinases Promotes Hedgehog Signaling. *Dev Cell* 2019; **50**(5)**:** 610-626.
16. Collado-Torres L, Burke EE, Peterson A, Shin J, Straub RE, Rajpurohit A *et al.* Regional Heterogeneity in Gene Expression, Regulation, and Coherence in the Frontal Cortex and Hippocampus across Development and Schizophrenia. *Neuron* 2019; **103**(2)**:** 203-216.
17. Walker RL, Ramaswami G, Hartl C, Mancuso N, Gandal MJ, De La Torre-Ubieta L *et al.* Genetic Control of Expression and Splicing in Developing Human Brain Informs Disease Mechanisms. *Cell*

2019; **179**(3)**:** 750-771.

18. Jiang Y, Qian FC, Bai XF, Liu YJ, Wang QY, Ai B *et al.* SEdb: a comprehensive human super-enhancer database. *Nucleic Acids Res* 2019; **47**(D1)**:** D235-D243.

The unmodified EMSA blots

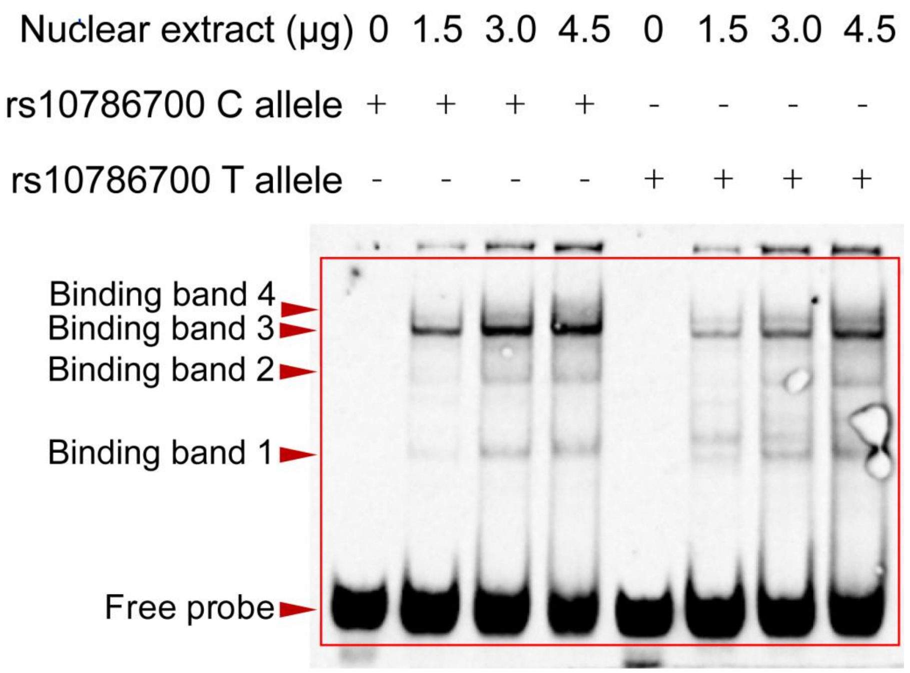

Original EMSA blots of Fig. 2I

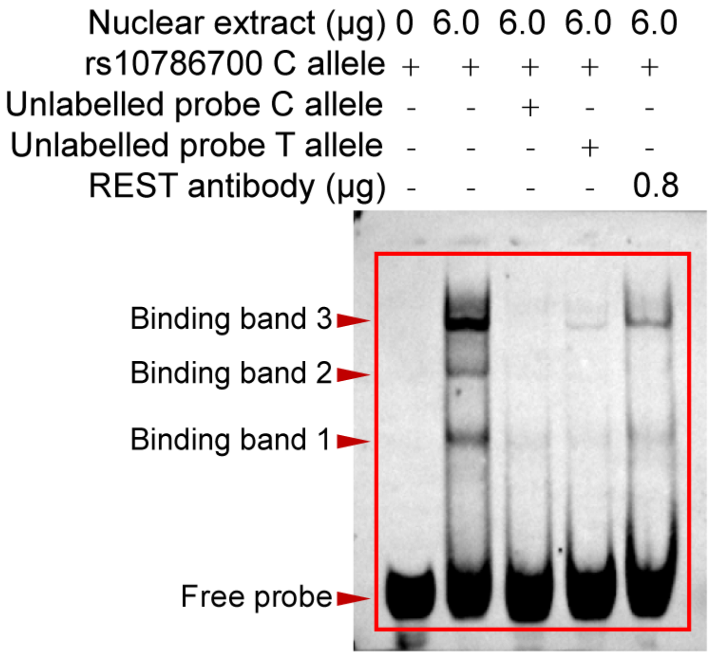

Original EMSA blots of Fig. 2J

The unmodified EMSA blots

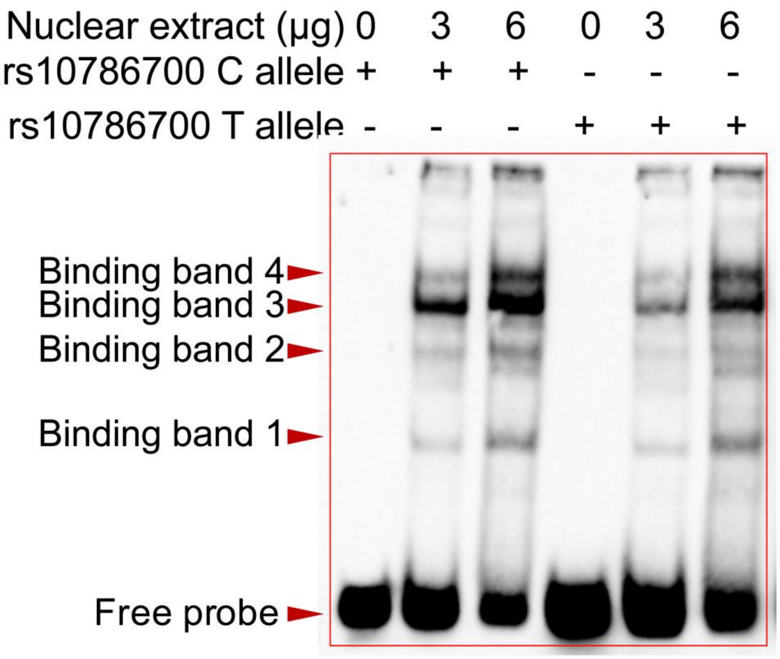

Original EMSA blots of Fig. 3C

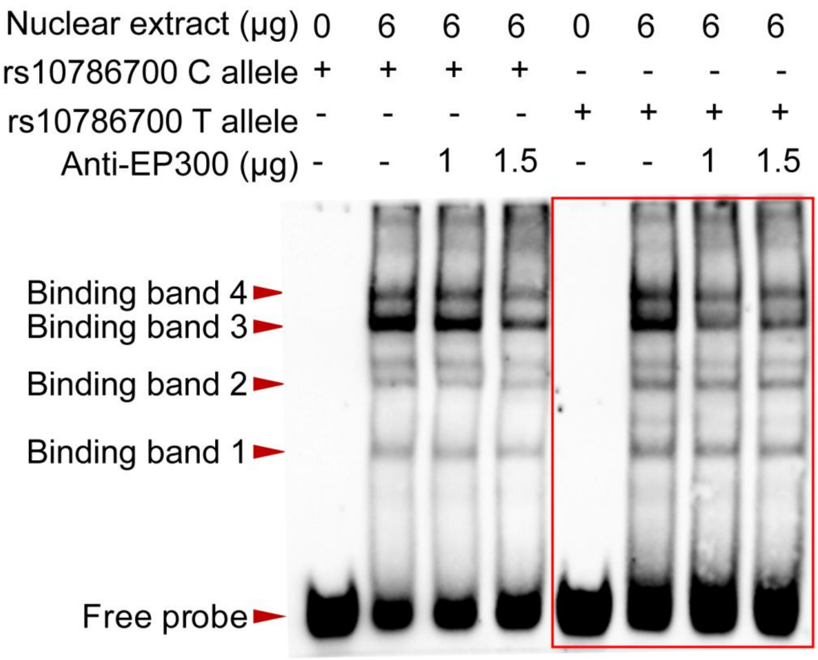

Original EMSA blots of Fig. 3D

The unmodified gels of PCR

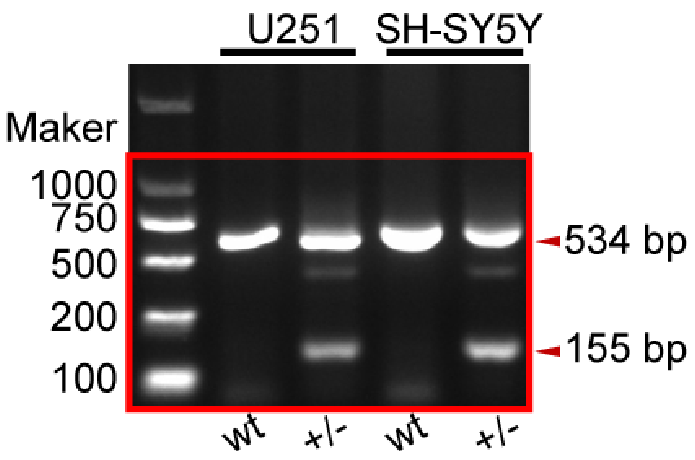

Original gels of Fig. 3E

The unmodified blots of Western-blot

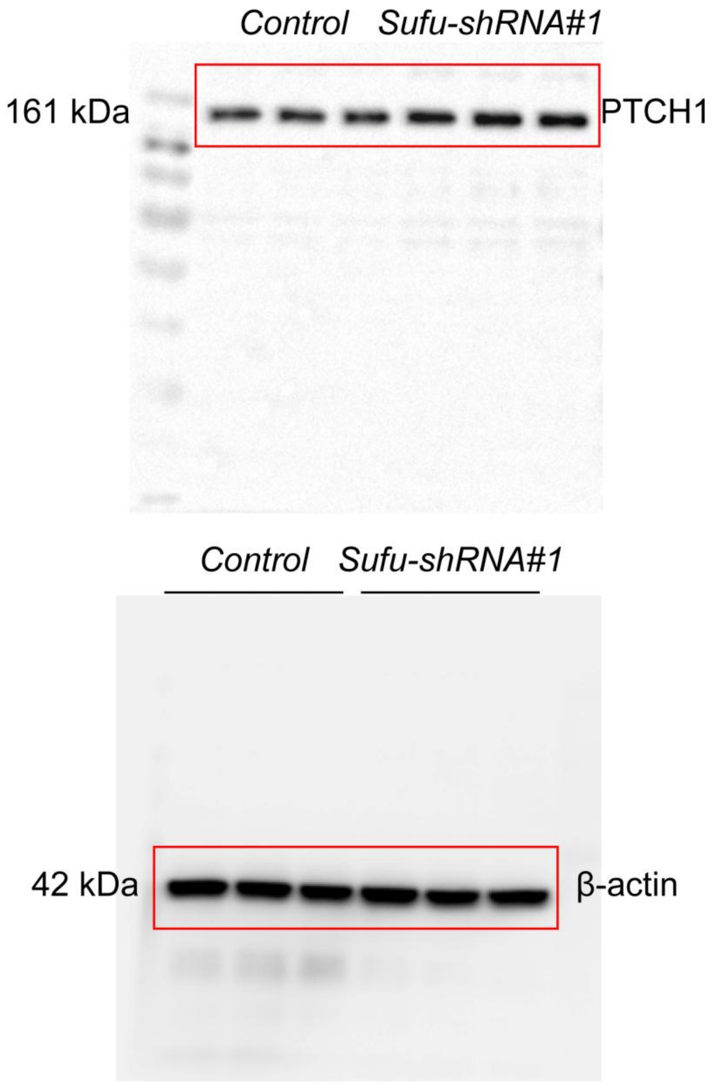

Original blots of Fig. 5F
